# One-dimensional palladium MOF as VEGFR2 and colchicine binding inhibitors with potential anticancer and anti-inflammatory activities: synthesis and molecular investigation

**DOI:** 10.1038/s41598-026-63591-z

**Published:** 2026-07-30

**Authors:** Heba K. Abdelhakim, Safaa S. Hassan, Khaled M. Ismail

**Affiliations:** 1 Biochemistry Division, Chemistry Department, Faculty of Science, Cairo University, Cairo, Egypt; 2 Chemistry Department, Faculty of Science, Cairo University, Giza, 12613 Egypt

**Keywords:** 1D Pd MOF, VEGFR2 kinase, Apoptotic genes, Tubulin-interacting drugs, DFT and docking, Biochemistry, Cancer, Drug discovery, Oncology

## Abstract

The innovation of a therapeutic agent with dual anti-inflammatory, anticancer and reversing the dynamic of microtubules like colchicine was crucial. VEGFR2 inhibition has been established as a therapeutic approach for managing cancer, the colchicine site, situated on ß-tubulin and α-tubulin was also considered in cancer development, and metastasis. Moreover, uncontrolled inflammation predisposes to pleiotropic effects leading to cancer development and promoting all stages of tumorigenesis. A polymeric state of a novel one-dimensional palladium-based metal-organic framework (1D Pd-MOF), resulting from the coordination of pyrazine with palladium nuclei to achieve the more pronounced effect of polynuclear characteristics of palladium compared to mononuclear compounds, was designed, synthesized, and screened for its anticancer activity against the A549 lung cancer cell line, with comparison to normal cells. The characterization was performed using different techniques and supported by DFT investigations. The 1D Pd MOF exhibited a potent cytotoxic effect, with an IC_50_ value of 78.21 ± 0.41 µg/mL against the A549 lung cancer cell line, while showing minimal toxicity toward the normal WI-38 cell line. To investigate the inhibitory activity of the 1D Pd-MOF towards VEGFR2 kinase and to confirm itseffective molecular target, VEGFR2 kinase inhibition was evaluated. The results demonstrated promising VEGFR2 inhibitory activity with a moderate IC_50_ value of 0.658 ± 0.023 µg/mL, compared with an IC_50_ value of 0.079 ± 0.003 µg/mL for sorafenib, the reference drug. This activity was further supported by significant inhibition of VEGFR2 gene expression and protein levels compared with untreated control cells. Our results revealed that 1D Pd-MOF is a promising tubulin-interacting compound, as it binds to the colchicine-binding site on tubulin and competes with colchicine in vitro, with an IC_50_ value of 3.350 ± 0.21 µg/mL. These findings confirm tubulin as a molecular target of the 1D Pd-MOF and support its observed cytotoxic activity. Furthermore, the 1D Pd-MOF significantly upregulated the expression levels of BAX, P53, and caspase-3 genes, while downregulating CDK4, cyclin D1, and BCL2 genes relative to the control group, suggesting growth inhibition and induction of apoptosis in treated lung carcinoma cells. These findings confirm that the 1D Pd-MOF exerts a clear anticancer effect by triggering programmed cell death through apoptosis. In addition, treatment with the 1D Pd-MOF resulted in substantial accumulation of cells in the sub-G1 fraction, accompanied by a corresponding reduction in the G2/M phase, indicating that its cytotoxic efficacy is primarily mediated through apoptosis induction rather than cell cycle arrest at a specific checkpoint. The anti-inflammatory activity of the 1D Pd-MOF was established by its inhibition of nitric oxide (NO) production in LPS-stimulated RAW cells. Molecular docking studies showed good agreement with the experimental biological findings.

**Supplementary Information:**

The online version contains supplementary material available at 10.1038/s41598-026-63591-z.

## Introduction

Tumor treatment strategies include promoting cell death, delaying signal transduction, enhancing gene modulator expression, and inhibiting angiogenesis^1,2^. Apoptosis is a gene-regulated, energy-dependent process that initiates programmed cell death, serving as a protective mechanism that helps organisms adapt to their environment^3^. Disruption of apoptotic pathways can lead to carcinogenesis by upsetting the balance between cell growth and death, resulting in uncontrolled proliferation^4^. Therapeutic agents that induce apoptosis by targeting key regulatory proteins (such as BAX, BCL-2, caspase-3, caspase-9, tubulin, and various kinases) hold significant promise for effective tumor control and cancer treatment strategies^5^. Apoptosis is activated by upregulation of BAX and downregulation of BCL-2^6^, with caspases (both initiator and effector) playing key roles in the cascade^7^. Among vascular endothelial growth factor (VEGF) receptors, VEGFR1, VEGFR2, and VEGFR3 regulate apoptosis, with VEGFR2 being the most relevant in tumor-related apoptosis, making it a critical therapeutic target^8–12^. Tyrosine kinase inhibitors (TKIs) play a central role in cancer therapy by inhibiting the VEGF/VEGFR2 signaling pathway, thereby blocking downstream signal transduction^13^. Tumor cells promote angiogenesis by releasing VEGF, which activates VEGFR2 on endothelial cells and contributes to tumor progression^14^. Although many VEGFR2-related clinical trials have been documented^15^, only a few small molecules, such as sorafenib, have received FDA approval as effective VEGFR2 inhibitors^16^.

The microtubule system, an essential component of the eukaryotic cytoskeleton, plays a key role in mitotic spindle formation and cell division^17–20^. Novel molecules are being developed to disrupt microtubule dynamics, which are critical for cancer cell proliferation^21–23^. Colchicine site binders-tubulin-interacting agents developed from both natural and synthetic sources-target the colchicine-binding site located on α- and β-tubulin^24–27^, making this site a valuable target in tumor growth, progression, and vascularization^28,29^.

Inflammation is a recognized hallmark of cancer, contributing to tumor progression through acute and chronic responses^30–32^, and is linked to over 15% of cancer-related deaths globally^33,34^. The complexity of cancer types demands more specific and selective therapies, which current treatments often lack^35,36^. NSAIDs like aspirin, lonazolac, and celecoxib have been shown to reduce cancer risk and mortality associated with neoplastic transformation^11,37–39^, but their long-term use can cause serious side effects^38,40–44^. As a safer alternative, dual PGE2/iNOS inhibitors offer promise by reducing excessive PGE2 and NO production in inflammatory conditions^40,45–47^.

Nanomedicine has the potential to help with the early identification and treatment of cancer because of its therapeutic capabilities, active or passive targeting, high solubility, biocompatibility, bioavailability, and multifunctionality, which are all advantages that make it superior to conventional cancer therapies^48–50^.

Nitrogen-based heterocycles, particularly pyrazines, have gained attention for their role in drug design and discovery^51^. Pyrazines, which contain two nitrogen atoms in their aromatic ring, serve as intermediates in pharmaceuticals, agricultural chemicals, and fragrances^5,6^. Pyrazine derivatives exhibit potent anticancer activity and have been shown to inhibit a wide range of enzymatic targets, including protein kinases, mitotic kinases, BRAF, mTOR complexes, checkpoint kinases, and caspases, particularly in A549 lung cancer cells^52–54^.

Transition metals, especially palladium, are increasingly used as the core of cytostatic agents due to their chemical similarity to platinum. Both belong to the Pt-group elements (PGE) and form bonds of comparable lengths. Pd complexes interact with cancer cell DNA through both covalent and non-covalent mechanisms, including hydrogen bonding and electrostatic interactions. Unlike cisplatin, Pd(II) compounds bind to oligonucleotides such as [d(CGCGAATTCGCG)]_2_, blocking DNA replication and overcoming drug resistance. These complexes induce apoptosis through both intrinsic (mitochondrial) and extrinsic (death receptor-mediated) pathways. Mechanistically, they increase Bax expression, suppress Bcl-2, reduce mitochondrial membrane potential, and release cytochrome c to activate the caspase cascade. In the extrinsic pathway, Pd(II) enhances DR4 and DR5 gene expression. Additionally, they cause endoplasmic reticulum (ER) stress through reactive oxygen species (ROS) generation, which results from interactions with thiol-containing antioxidant proteins like GST, GPxs, CAT, and GSH, leading to their depletion and cellular damage. G2/M phase cell cycle arrest is also observed^55,56^. Pd compounds have shown lower toxicity than their Pt counterparts up to tenfold in rat studies^57^. This reduced toxicity may be attributed to the inability of sulfhydryl groups to replace the strongly bound chelating ligands of Pd(II) inside cells^58^.

The current study was designed to synthesize a novel compound by combining pyrazine and Pd nuclei, aimed at forming a polymeric structure. This design leverages the polynuclear nature of Pd to enhance anticancer efficacy over mononuclear compounds. The compound was evaluated for its activity against the A549 lung cancer cell line through proliferation assays, tyrosine kinase inhibition, and colchicine site binding. Mechanistic studies included various apoptotic assays to explore its dual anticancer and anti-inflammatory potential. Molecular docking was conducted to confirm the interaction of the compound with the active sites of target proteins.

## Experimental

### Materials and methods

#### Materials

Palladium (II) chloride (PdCl_2_), potassium chloride (KCl), and pyrazine (Pyz) were obtained from Sigma-Aldrich and used without further purification.

#### Synthesis of 1D-Pd-MOF

To prepare the tetrachloropalladate (II) complex [PdCl_4_]^2−^, PdCl_2_ (1.0 mmol) and KCl (2.0 mmol) were dissolved in a minimal amount of distilled water and heated to 70 °C under constant stirring. The resulting solution was then mixed with an equimolar solution of pyrazine (1:1 molar ratio) to facilitate the formation of a one-dimensional palladium-based metal-organic framework (1D Pd-MOF) as previously reported by Safaa et al.^59^.

A brown precipitate formed immediately upon mixing, which was collected by filtration, washed thoroughly with distilled water to remove any unreacted species or byproducts, and then dried at room temperature. The final product, identified as [Pd(Pyz)Cl_2_]_n_, was obtained as a brown solid.

Elemental Analysis: Found – C: 27.99%, H: 2.18%, N: 16.09%; Calculated – C: 28.47%, H: 2.39%, N: 16.60%; Yield: 75.0%.

### Biological evaluation

#### Antiproliferative activity by MTT assay

See supplementary file.^60^

#### VEGFR2 kinase inhibitory assay

The inhibitory activity of the compound against VEGFR-2 kinase was evaluated using an Alpha Screen assay (PerkinElmer, USA) based on phosphotyrosine antibody detection, according to the manufacturer’s protocol. The enzymatic reaction was conducted in a buffer containing 50 mM Tris-HCl (pH 7.5), 5 mM MnCl_2_, 0.5 mM MgCl_2_, 0.01% Tween-20, and 2 mM DTT. The reaction mixture included 10 mM ATP, 0.1 mg/mL biotinylated poly-Glu: Tyr (4:1), and 0.1 nM VEGFR-2 enzyme (Millipore, UK).

The compound was tested at concentrations ranging from 0.01 to 100 µg/mL. It was pre-incubated with the enzyme for 5 min at room temperature before initiating the reaction with ATP. The reaction was stopped by adding 25 µL of 100 mM EDTA and AlphaScreen donor and acceptor beads (each at 10 mg/mL) prepared in 62.5 mM HEPES (pH 7.4), 250 mM NaCl, and 0.1% BSA. Plates were incubated overnight in the dark, and the signal was detected using an ELISA reader (PerkinElmer, USA).

Control wells included enzyme and substrate without the test compound (positive control) and enzyme with substrate but without ATP (negative control). Percent inhibition was calculated relative to the control wells. IC_50_ values were determined from dose–response curves (based on triplicate measurements) and compared with those of Sorafenib, a known VEGFR-2 inhibitor^61,62^ In addition, the concentration of VEGF Receptor 2 (VEGFR2) was measured utilizing a commercial Human VEGF Receptor 2 SimpleStep ELISA^®^ Kit (Abcam, Cambridge, UK, Cat. No. ab213476) in accordance with the manufacturer’s guidelines^63^. The concentrations were determined by regression analysis relative to the established standard curve. All experiments were conducted in duplicate, and the data are presented as the mean ± standard deviation (SD). Statistical significance was assessed using an unpaired t-test conducted with GraphPad Prism software, and *p* < 0.05 was deemed statistically significant.

#### [^3^h] colchicine–tubulin binding assay

A colchicine-binding assay was performed using 1 µM [ring C, methoxy-^3^H]-radiolabeled colchicine (PerkinElmer), 1% DMSO, and varying concentrations of the test compound in 50 µL of G-PEM buffer (80 mM PIPES, pH 6.8, 1 mM EGTA, 1 mM MgCl_2_, 1 mM GTP, and 5% glycerol). This mixture was incubated with 1 µM purified tubulin (> 99% purity; Cytoskeleton, Inc., final concentration 0.2 mg/mL) at 37 °C for 60 min.

After incubation, the reaction mixtures were filtered through a double-layered DEAE-cellulose filter stack and washed twice with cold buffer. The retained radioactivity on the filters was quantified using liquid scintillation counting (PerkinElmer Wallac). Data were analyzed using nonlinear regression in GraphPad Prism to determine binding parameters^64^.

#### Inhibitory activity of NO over-production

See supplementary file^44^.

#### Assessment of the apoptotic cell death by Pd MOF

##### Cell cycle analysis

The procedure for this assay was conducted in accordance with previous studies^65,66^. A total of 3 × 10^6^ cells were seeded into two culture flasks and incubated for 48 h. Cells were then treated with the IC_50_ concentration of the test compound, 1D Pd-MOF. After 48 h of treatment, single-cell suspensions were prepared by trypsinization using a trypsin/EDTA solution in RPMI medium. Detached cells present in the culture medium were also collected for analysis. Cell suspensions (1 × 10^6^ cells) were centrifuged, resuspended in 200 µL of phosphate-buffered saline (PBS), and fixed in 2 mL of 70% ice-cold ethanol at 4 °C for at least 30 min. Following fixation, cells were washed twice with PBS and resuspended in 800 µL PBS containing 100 µL of RNase A (1 mg/mL) and 100 µL of propidium iodide (PI, 400 µg/mL). The mixture was incubated at 37 °C for 30 min. Flow cytometric analysis was performed using an Epics XL-MCL flow cytometer (Beckman Coulter, Miami, FL). Cell cycle distribution was analyzed using MultiCycle software (Phoenix Flow Systems, San Diego, CA), and the percentages of cells in the G0/G1, S, and G2/M phases were quantified. All experiments were conducted in triplicate, and data are presented as mean values.

##### Annexin V apoptosis assay

Apoptosis was assessed using the Annexin V–FITC apoptosis detection kit following the manufacturer’s instructions. A total of 3 × 10^6^ A549 cells were seeded into four culture flasks, two designated as controls and two for treatment, and incubated for 48 h. Treated cells were exposed to 78.21 µg/mL of the test compound, Pd-MOF, for 48 h. After incubation, single-cell suspensions were prepared by trypsinization using a trypsin/EDTA solution in RPMI medium. Cells that had detached naturally into the culture medium were also collected and included in the analysis.

Cell suspensions (1 × 10^6^ cells) were centrifuged at 500 g for 5 min at 4 °C, washed with PBS, and resuspended in ice-cold 1× binding buffer at a concentration of 5 × 10^6^ cells/mL. To each 100 µL of cell suspension, 5 µL of PI and 1 µL of Annexin V–FITC solution were added and gently mixed. Samples were incubated on ice for 15 min in the dark. After incubation, 400 µL of ice-cold 1× binding buffer was added to each tube and mixed gently. Samples were analyzed within 30 min using flow cytometry. Each experiment was repeated three times, and the results were expressed as the mean values^67^.

##### Real-time-polymerase chain reaction for the selected genes

See supplementary file^68^.

##### Estimation of the level of Cyclin D1 by ELISA

The protein expression levels of human Cyclin D1 were quantified using a commercial solid-phase sandwich enzyme-linked immunosorbent assay (ELISA) kit (Catalog No. MBS724349, MyBioSource, Inc., USA) according to the manufacturer’s instructions^69^. The concentrations of Cyclin D1 in the samples were determined by interpolation from the standard curve. All experiments were conducted in duplicate, and the data are presented as the mean ± standard deviation (SD). Statistical significance was assessed using an unpaired t-test conducted with GraphPad Prism software, and *p* < 0.05 was deemed statistically significant.

##### Acridine orange/ethidium bromide double staining assay

The morphological characteristics of apoptosis in treated A549 cells were evaluated using the acridine orange/ethidium bromide (AO/EB) dual staining method. A549 cells were cultured on 8-well cell culture slides and incubated overnight. The following day, the cells were treated with the 1D Pd-MOF compound at a concentration equivalent to 1/2 IC_50_ for 48 h. Control cells were cultured under identical conditions but without exposure to the compound. After the treatment period, both treated and control cells were harvested. The cell suspensions were mixed with an equal volume of AO/EB staining solution, prepared in phosphate-buffered saline (PBS) and containing 100 µg/mL of acridine orange and 100 µg/mL of ethidium bromide. The stained cells were immediately examined under a fluorescence microscope (Axio Imager Z2, Zeiss, Jena, Germany) to visualize apoptotic features, including chromatin condensation, membrane blebbing, and nuclear fragmentation^70^.

### Statistical analysis

Statistical analysis was performed using GraphPad Prism 8 software (GraphPad Software, Inc., La Jolla, CA, USA). The data are presented as the mean ± standard deviation (SD). p-values < 0.05 were considered statistically significant. All experiments were conducted in triplicate to ensure the reproducibility of the results.

### Computational study

See supplementary file^71^.

## Results and discussion

### Characterization

#### Experimental and theoretical DFT investigations

The formation of the 1D Pd-MOF was confirmed using various analytical and spectroscopic techniques.

##### Elemental analysis and conductivity

The elemental analysis data (provided in the Experimental section) showed excellent agreement with the theoretical values, supporting the proposed molecular composition. The molar conductivity of the 1D Pd-MOF in anhydrous DMF was found to be significantly low, indicating a non-electrolytic nature^72^. This suggests that chloride ions are directly coordinated to the palladium center rather than existing as free counterions, confirming the formation of a neutral coordination compound.

##### Infrared spectroscopy (FTIR)

**Fig. 1:**
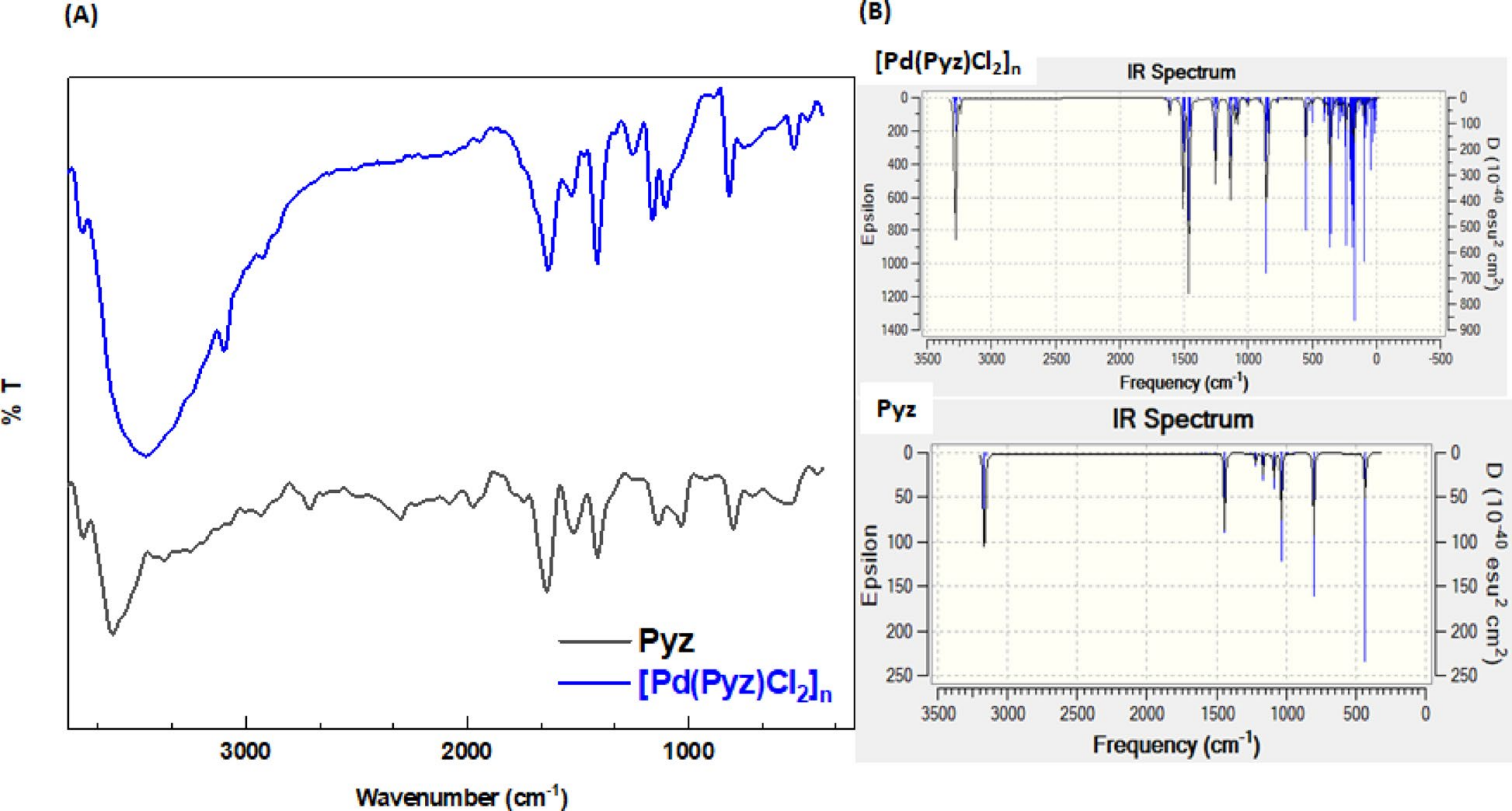
(**A**) Experimental and (**B**) Simulated of FTIR spectra of pyrazine (Pyz) and [Pd(Pyz)Cl_2_]_n_.

The IR spectra of free pyrazine (Pyz) and its 1D Pd-MOF are compared in Table 1; Fig. 1A, with key band assignments provided. For the free ligand, bands at 3086 cm^−1^ (νCH), 1651 cm^−1^ (νC = N), 1327 cm^− 1^ (νC-N) and 1527 cm^−1^ (νC = C) were observed^73,74^. Upon coordination with Pd(II), the νC = N band shifts to 1643 cm⁻¹, indicating that the nitrogen atoms of the pyrazine ring are involved in coordination with the metal center. Similarly, the νCH stretch shifts slightly to 3093 cm^−1^, and a prominent band at 1149 cm⁻¹ (pyrazine ring vibration) shifts to 1157 cm^−1^, further confirming ring participation in coordination. A distinct metal-ligand bond signal appears at 532 cm^−1^, which is attributed to the Pd–N stretching vibration^75^.

Theoretical IR spectra of the free Pyz ligand showed characteristic peaks at 3166, 1614, and 1511 cm^−1^, corresponding to νCH, νC = N, and νC = C vibrations, respectively. These bands were also observed to shift after coordination with Pd, in alignment with the experimental results. Overall, the simulated FTIR data exhibit strong correlations with experimental observations, as shown in Fig. 1B and detailed in Table 1.

**Table 1:** Infrared spectral data of “Pyz” and its Pd (II) complex (cm^− 1^).

Vibrational frequency (cm^− 1^)				Assignment
Pyz		[Pd(Pyz)Cl_2_]_*n*_		
Exp.	Theo.	Exp.	Theo.	
3070	3166	3093	3240	*ν*CH
1527	1511	1527	1503	*ν*C = C
1643	1614	1635	1608	*ν*C = N
–	–	524	548	*ν*Pd-N

##### Mass spectrometry

**Fig. 2:**
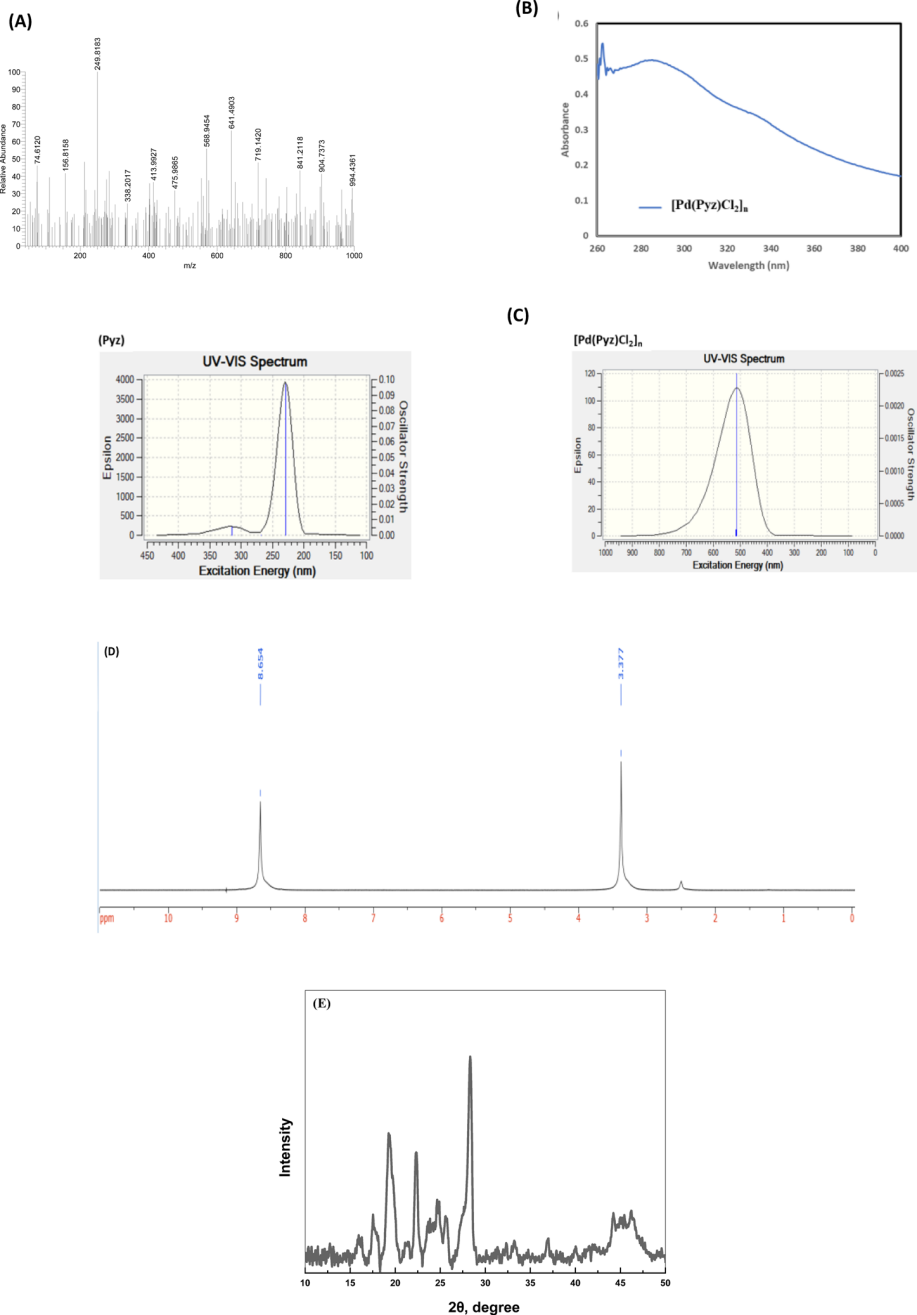
(**A**) Mass spectrum of [Pd(Pyz)Cl_2_]_n_, (**B**) Experimental electronic transitions of [Pd(Pyz)Cl_2_]_n,_ (**C**) Simulated of electronic transitions of pyrazine (Pyz) and [Pd(Pyz)Cl_2_]_n_, ¹H NMR chart of [Pd(Pyz)Cl_2_]_n_ and PXRD of [Pd(Pyz)Cl_2_]_n_.

The mass spectrum (Fig. 2A) of the synthesized compound revealed a wide range of fragment ions, with values extending to the upper detection limit of the instrument. This confirms that the compound possesses a high molecular weight, exceeding 1000 amu, consistent with a polymeric structure. The fragmentation pattern was represented in Scheme S1 that may be initiated by cleavage of the coordination polymer chain, generating mononuclear and oligonuclear palladium-containing species while partially preserving the Pd–pyrazine coordination environment. Subsequent fragmentation involves cleavage of Pd–N coordination bonds, partial dechlorination, and dehydrogenation of the pyrazine ligands, giving rise to the characteristic fragment ions observed at m/z 249.01, 337.50, 408.47, 475.37, 567.91, 641.49, 904.59, and 994.44. The base peak at m/z 249.01 is assigned to the most stable mononuclear palladium species formed following polymer chain dissociation, whereas the higher m/z ions are consistent with fragments containing two or more Pd–pyrazine repeating units. In addition, the deprotonated ion at m/z 904.59 is consistent with ligand dehydrogenation during the ionization process. The proposed fragmentation pathway is consistent with the expected behavior of a coordination polymer containing bridging pyrazine ligands and complements the spectroscopic, microscopic, and computational evidence supporting the proposed one-dimensional Pd-MOF structure.

##### UV-visible spectroscopy

UV-Vis spectra of the free Pyz ligand and the 1D Pd-MOF were recorded at room temperature (Fig. 2B). The free pyrazine displays absorption bands between 220 and 270 nm and beyond 290 nm, which are attributed to π–π* transitions within the aromatic system and n–π* transitions of the C = N moiety, involving lone pair electrons on nitrogen^76,81[76-81]^. Upon coordination, these bands are red-shifted to 261, 286.5, and 335 nm, respectively. The new band at 335 nm is likely due to ligand-to-metal charge transfer (LMCT), indicating strong interaction between the ligand and the metal center.

##### Theoretical UV-Vis analysis

Simulated electronic transitions (Fig. 2C) for the free Pyz ligand appear at 230 nm and 314 nm, which closely resemble the experimental values. For the 1D Pd-MOF, a significant band appears at 515 nm, consistent with experimental observations. Minor differences between theoretical and experimental values may arise from the differences in the gas-phase vs. solution-phase environments, or from limitations in the calculated chain length used in the TD-DFT method.

##### ^1^H NMR spectrum

Experimental spectra of pyrazine recorded in organic solvents indicate a singlet around δ ≈ 8.59 ppm for the ring protons of free C_4_H_4_N_2_^82^. In the ^1^H NMR spectrum of [Pd(pyz)Cl_2_]_n_ measured in DMSO-d₆, the dominant resonance observed at ~ 8.65 ppm as seen in Fig. 2D aligns closely with these values, confirming the presence of the aromatic pyrazine protons in a similar chemical environment but slightly shifted due to coordination to Pd(II). The absence of additional signals attributable to free pyrazine and the presence of consistent equivalent proton environments indicate a repeating, uniform coordination environment throughout the sample, which supports the formation of a coordination polymer rather than a mixture of free ligand and mononuclear species.

##### The X-ray diffraction (XRD)

In the presented diffractogram (Fig. 2E), the presence of several sharp and well-defined Bragg reflections clearly indicate that the sample possesses significant long-range crystalline order rather than being amorphous—amorphous materials typically show only broad diffuse features without sharp peaks^83–85^. The PXRD pattern of PdCl_2_ shows characteristic reflections such as (002) at 16.12° 2θ and (101) at 24.70° 2θ (COD: 1010447), while the pure pyrazine ligand exhibits its own distinct peaks including the (110) plane at 19.0° 2θ and (10 − 1) at 26.5° 2θ (COD: 14215015). In contrast, the diffractogram of [Pd(pyrazine)Cl_2_]_n_ reveals a unique set of diffraction peaks at 15.92°, 22.33°, 24.64°, 31.36°, 36.95°, and 40.04° 2θ that do not directly match the patterns of either precursor, indicating the formation of a new crystalline phase. These reflections, indexed to specific Miller planes, arise from the periodic arrangement of Pd(II) centers coordinated to chloride ligands and pyrazine bridges according to Bragg’s law, reflecting long-range order in the extended polymeric structure. The shift and re-organization of peak positions relative to those of PdCl_2_ and pyrazine confirm that the coordination of the pyrazine ligand to PdCl_2_ alters the unit cell parameters and crystal symmetry, further validating the successful synthesis of the targeted coordination polymer rather than a simple physical mixture of starting materials.

##### Magnetic moment

The magnetic moment of the 1D Pd-MOF was measured to be 0.0 B.M., which confirms its diamagnetic nature. This result is consistent with a square planar geometry around the Pd(II) center^86,87^, which is typical for d^8^ metal compounds.

##### Optimization, frontier molecular orbitals, molecular electrostatic potential and reactivity parameters

**Fig. 3:**
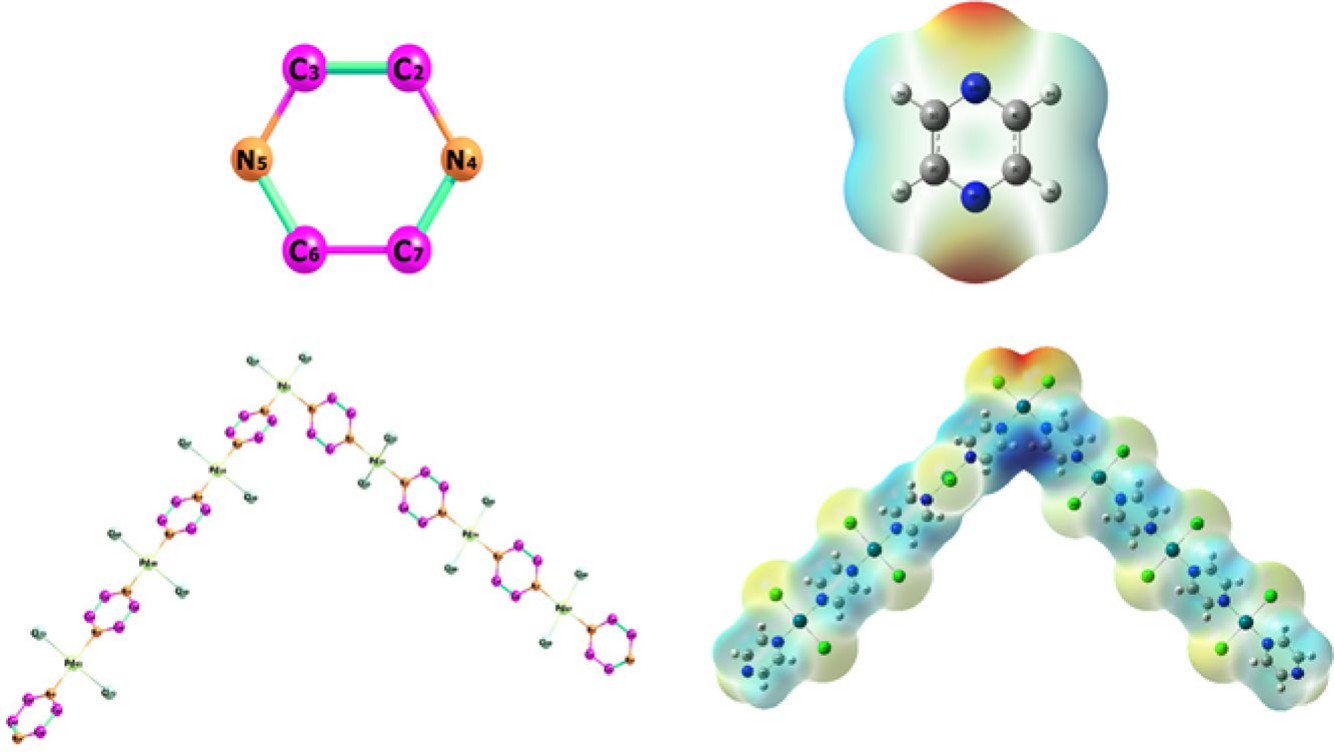
Geometric structure and Mep of Pyz linker and Pd polymeric complex.

To represent the polymeric structure, DFT calculations were performed using a finite chain fragment extracted from the proposed 1D Pd-MOF as illustrated in Fig. 3. This model preserves the local coordination environment of the Pd(II) center and the bridging mode of the pyrazine ligands, thereby representing the essential structural motif of the one-dimensional coordination polymer. The Pd(II) center is coordinated to four atoms: two nitrogen atoms (N5 and N11) originating from two adjacent bridging pyrazine ligands and two chloride ions (Cl14 and Cl15), forming a square planar coordination environment. The calculated bond angles around the Pd center support this geometry, with values such as Cl14–Pd–Cl15 = 86.98° and N5–Pd–N11 = 93.25°, which are close to the ideal 90° typically observed in square planar coordination. Minor adjustments in bond lengths were observed in the C = N bonds of the pyrazine ring upon coordination, as detailed in Table S2, to accommodate the Pd–N interaction.

The Molecular Electrostatic Potential (MEP) map (Fig. 3) provides insight into the charge distribution across the molecule. In the MEP diagram, red regions represent areas of high electron density (electrophilic attack sites), while blue regions indicate areas of low electron density (nucleophilic attack sites). For the free pyrazine linker, the electron-rich regions are localized over the nitrogen donor atoms (N4 and N5), highlighting them as preferred coordination sites. After coordination, the electron distribution changes slightly, indicating interaction with the Pd(II) center.

Using Natural Bond Orbital (NBO) analysis, the natural charges on the coordinating nitrogen atoms (N4 and N5) were calculated. In the free ligand, both N4 and N5 exhibited identical negative charges of − 0.41453. Upon coordination, these values shifted to − 0.46364, indicating an increase in electron density due to Pd–N bonding. Simultaneously, the palladium ion acquired a positive natural charge of + 0.35442, consistent with its role as a central electron-accepting metal in the compound^88^.

The ground-state electronic properties of both the free pyrazine ligand and its 1D Pd-MOF were evaluated using quantum chemical descriptors, with values summarized in Table 2. The following standard equations were used to calculate key reactivity parameters:

Ionization potential (I) = – E_HOMO_; Electron affinity (A) = – E_LUMO_; Hardness (η) = (I – A)/2; Softness (S) = 1/(2η); Chemical potential (µ) = – (I + A)/2; Electronegativity (χ) = (I + A)/2.

**Table 2:** Some of the optimized bond lengths, Å and bond angles, degrees, Pyz linker and its Pd(II) chain using B3LYP/6-311G and B3LYP/LANL2DZ respectively.

Parameter	Pyz	Chain	Element	Pyz	Chain
				MPA(NPA)	
E_T_, Hartree	− 264.38	− 3211.54	**Pd1**	–	0.0963 (0.354)
E_HOMO_, eV	− 7.17	− 6.99	**C2**	− 0.0942 (0.011)	− 0.0830 (0.081)
E_LUMO_, eV	− 1.88	− 4.17	**C3**	− 0.0943 (0.011)	− 0.1107 (0.073)
Energy gap (ΔE, eV)	5.28	2.82	**N4**	− 0.0949 (− 0.414)	− 0.1831 (− 0.446)
I = − E_HOMO_, eV	7.17	6.99	**N5**	− 0.0949 (− 0.414)	− 0.1935 (− 0.463)
A = − E _LUMO_, eV	1.88	4.17	**C6**	− 0.0943 (0.011)	− 0.1090 (0.052)
Electronegativity (χ, eV)	4.53	5.58	**C7**	− 0.0943 (0.011)	− 0.0900 (0.076)
Hardness (η, eV)	2.64	1.41	**Cl14**	–	− 0.2291 (− 0.357)
Softness (S, eV^− 1^)	0.18	0.35	**C2**	− 0.0942 (0.011)	− 0.0830 (0.081)
Chemical potential (µ, eV)	− 4.53	− 5.58	**C3**	− 0.0943 (0.011)	− 0.1107 (0.073)
Electrophilicity [ω, eV (µ^2^ /2η)]	3.88	11.05	**N4**	− 0.0949 (− 0.414)	− 0.1831 (− 0.446)
Dipole moment (Debye)	0.0002	10.8173			

These parameters provide a theoretical basis for understanding the reactivity and stability of the compounds. The energy gap (E_HOMO_–E_LUMO_) is a critical indicator of chemical reactivity: a larger gap correlates with greater molecular stability and lower reactivity, while a smaller gap suggests increased reactivity^89^.

The free pyrazine ligand exhibited a larger energy gap compared to the 1D Pd-MOF, indicating higher stability. The reduced energy gap in the 1D Pd-MOF implies enhanced chemical reactivity, which may contribute to its potential biological activity. Additionally, the more negative total energy of the 1D Pd-MOF compared to the free ligand suggests that the coordination process is thermodynamically favorable.

The calculated electrophilicity index (ω) also provided insights into the electron-donating nature of the species. The free pyrazine displayed a lower ω value (Table 2), indicating better nucleophilic character compared to the metal-coordinated compound.

**Fig. 4:**
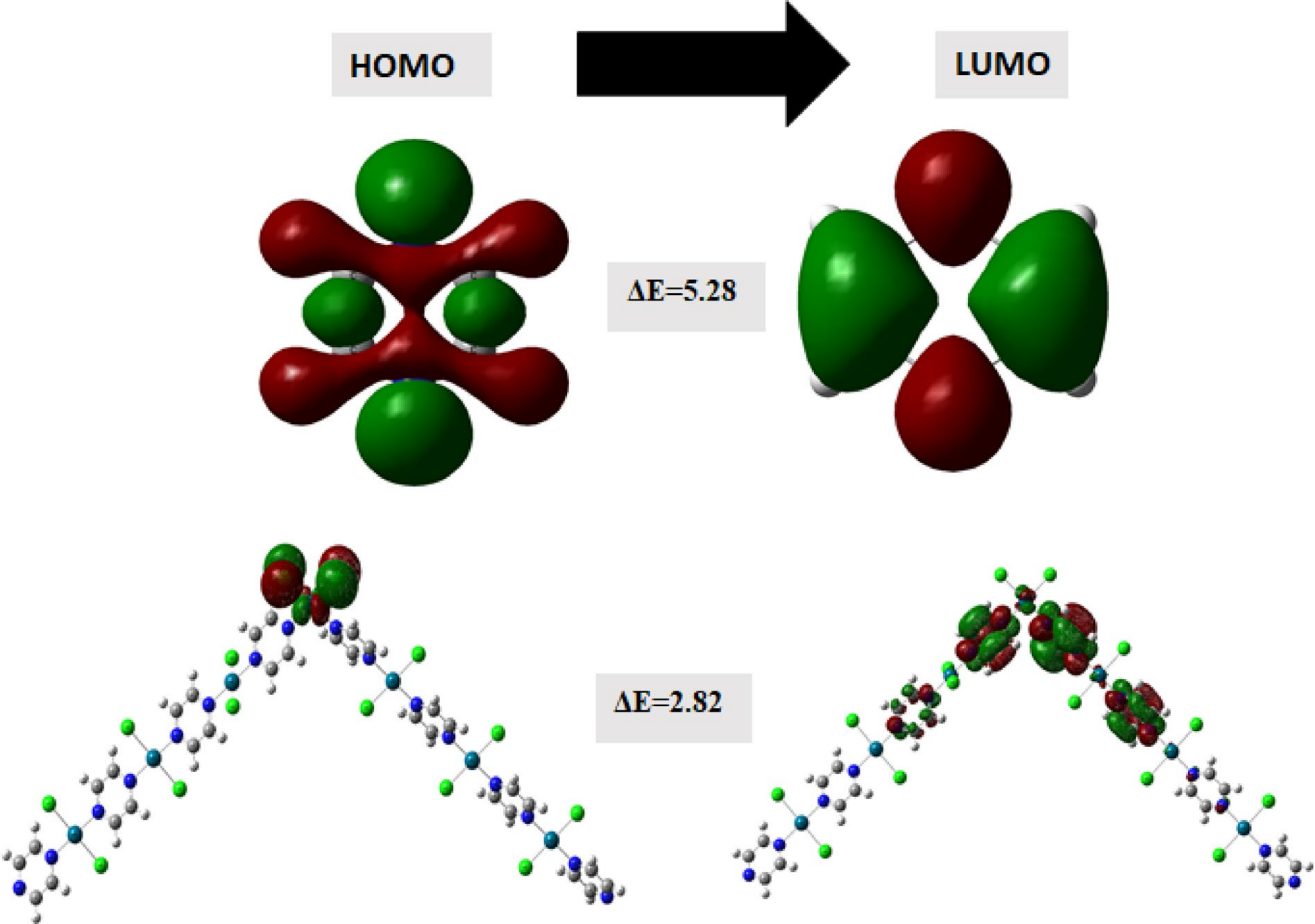
FMO of Pyz linker and Pd polymeric complex.

The spatial distributions of the HOMO and LUMO orbitals for both the free ligand and the Pd-MOF are presented in Fig. 4. These orbital diagrams help visualize electron delocalization and highlight the regions of the molecule involved in electron donation (HOMO) and acceptance (LUMO).

The calculated electronic properties provide a theoretical basis for the enhanced biological performance of the synthesized 1D Pd-MOF. The reduced HOMO–LUMO energy gap, together with the redistribution of electron density observed in the MEP map and NBO analysis, indicates increased electronic reactivity and the presence of favorable electron-rich and electron-deficient regions that may facilitate interactions with biological macromolecules. In particular, the localization of electron density around the coordinating atoms and Pd(II) centers suggests potential sites for intermolecular interactions, including metal-contact and donor–acceptor interactions with biomolecular targets. These theoretical findings provide a molecular basis for the experimentally observed enhanced biological activity and are further supported by the molecular docking results discussed later in the manuscript, in which the polymeric Pd-MOF exhibited stronger binding affinities toward the investigated protein targets than the corresponding monomer.

##### Morphological and elemental characterization

**Fig. 5:**
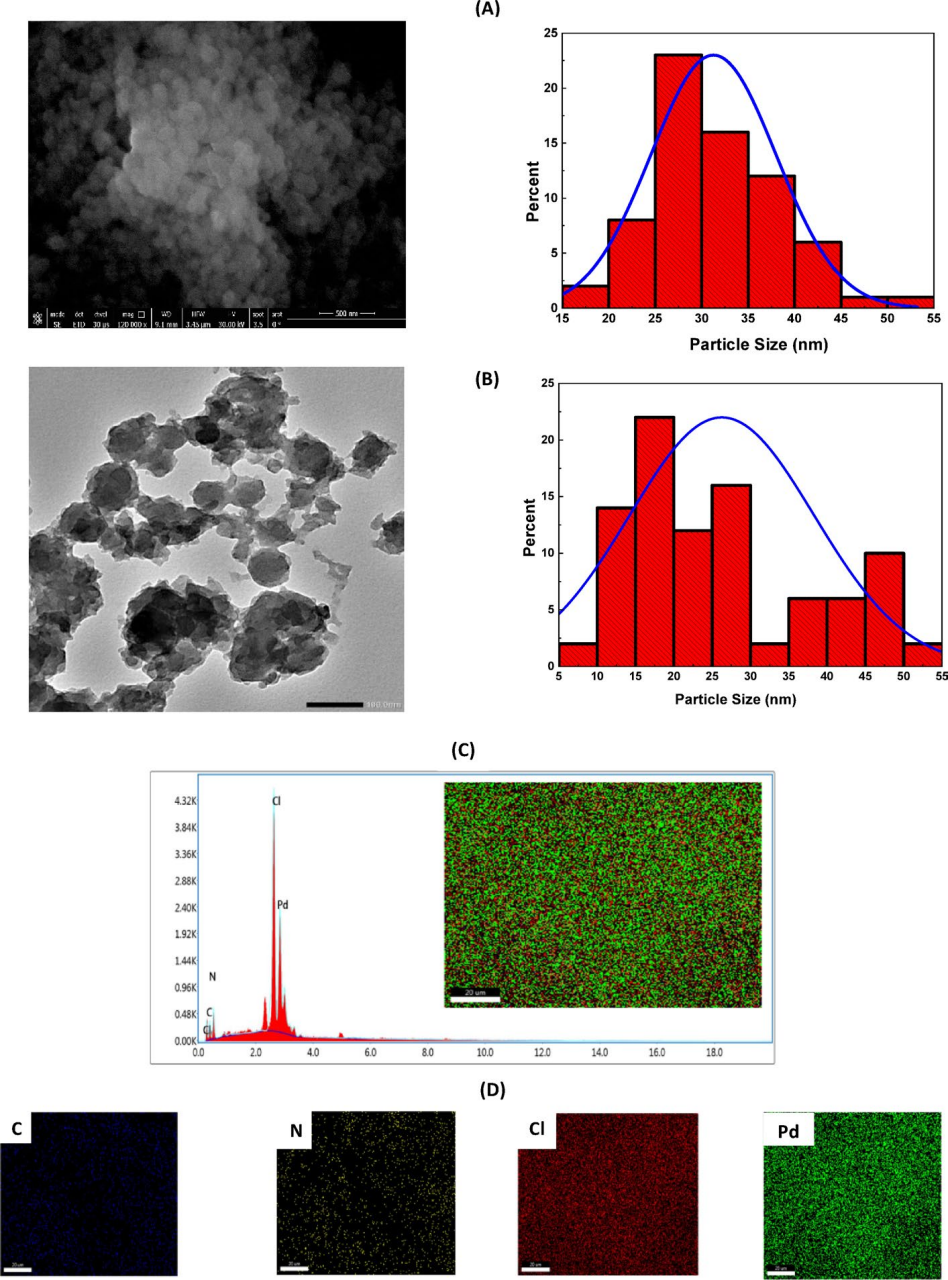
(**A**) SEM image of [Pd(Pyz)Cl_2_]_n_ and its particle size distribution, (**B**) TEM image of [Pd(Pyz)Cl_2_]_n_ and its particle size distribution, (**C**) EDX analysis, and (**D**) the elemental mapping of each element.

The morphology and elemental composition of the synthesized 1D Pd-MOF were examined using scanning electron microscopy (SEM), transmission electron microscopy (TEM), energy-dispersive X-ray spectroscopy (EDX), and elemental mapping, as presented in Fig. 5.

##### SEM and TEM analyses and particle size distribution

The SEM micrographs reveal that the 1D Pd-MOF consists of spherical particles, suggesting uniform crystal growth under the applied synthesis conditions. Understanding the particle size distribution is particularly important, as it can significantly influence the material’s surface area and biological activity, including cellular uptake and interactions with biological macromolecules. To analyze the particle size distribution, the diameters of individual particles were measured from high-resolution SEM images using ImageJ software. These measurements were statistically analyzed and presented as a histogram, which was then fitted with a Gaussian function. The resulting distribution indicated that the majority of the particles had diameters in the range of 25–37 nm, with an average particle diameter of 31.27 ± 6.74 nm (mean ± SD), as shown in Fig. 5A. The particle size analysis was further confirmed by transmission electron microscope (TEM), which revealed that the majority of particles had diameters in the range of 20–35 nm with an average particle diameter of 24.60 ± 10.84 nm (mean ± SD), as shown in Fig. 5B, consistent with the SEM results. This nanoscale size range is promising for biological applications.

#### EDX and elemental mapping

Elemental analysis using EDX spectroscopy (Fig. 5C) confirmed the presence of the key elements that make up the 1D Pd-MOF structure: carbon (C) and nitrogen (N) from the pyrazine ligand, chlorine (Cl) from the palladium coordination environment, and palladium (Pd) as the central metal ion. The elemental mapping results (Fig. 5D) further verify the homogeneous distribution of these elements across the entire surface of the particles, suggesting a uniform composition and successful integration of the ligand and metal components throughout the MOF structure.

These combined results from SEM, EDX, and elemental mapping provide strong evidence for the successful synthesis of a well-defined, nanostructured, and compositionally consistent 1D Pd-MOF material.

### Biological screening

#### Antiproliferative activity by MTT assay

A major challenge in cancer treatment is the ability of tumor cells to circumvent the therapeutic effects of chemotherapy. The antiproliferative effects of the newly synthesized Pd-MOF were assessed in the A549 lung cancer cell line and the non-cancerous WI-38 cell line. All experiments were conducted in duplicate. To ensure uniformity and facilitate comparative analysis, all treatments were performed for 48 h. Cells were treated with varying concentrations of Pd-MOF (31.25–1000 µg/mL) for 48 h.

The MTT assay was used to assess the inhibitory potential of Pd-MOF and determine the half-maximal inhibitory concentration (IC_50_). IC_50_ value represents the concentration required to inhibit 50% of cell viability. Pyrazine compounds demonstrate significant anticancer efficacy and have been proven to inhibit many enzymatic targets, including protein kinases. Pd(II) compounds, in contrast to their Pt counterparts, bind to specific oligonucleotides in DNA, thereby inhibiting DNA replication and circumventing drug resistance. Moreover, Pd compounds exhibit lower toxicity compared to their Pt counterparts. Pd-MOF exhibited significant cytotoxicity against A549 lung cancer cells, with an IC_50_ value of 78.21 ± 0.41 µg/mL. This selective cytotoxicity is particularly important, as it addresses a major limitation of conventional chemotherapeutic agents by reducing off-target toxicity^90^. The non-cancerous WI-38 cell line exhibited greater resistance to the cytotoxic effects of Pd-MOF, as indicated by a higher IC_50_ value of 163 ± 2.14 µg/mL. The selectivity index (SI) was calculated by dividing the IC_50_ value for WI-38 cells by the IC_50_ value for each cancer cell line. Pd-MOF exhibited a more favorable profile for A549 cells (SI = 2.08) than 5-FU (SI = 0.68).

This selectivity indicates that Pd-MOF may preferentially target A549 lung cancer cells while potentially minimizing damage to healthy, non-cancerous cells. Pd-MOF demonstrated negligible cytotoxicity towards the normal WI-38 fibroblast cell line, with no substantial toxicity observed at the evaluated doses.

**Fig. 6:**
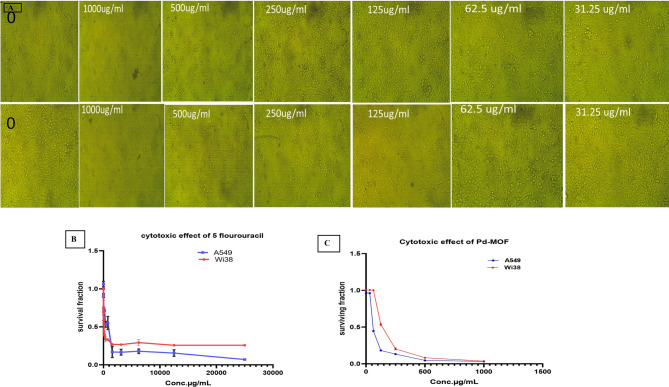
(**A**) Cytotoxic effect of Pd-MOF on A549 lung cancer cells (lower panel) and WI-38 normal lung fibroblast cells (Upper panel). (**B**) Dose-dependent reduction in cell viability following treatment with 5-fluorouracil (5-FU) and 1D Pd-MOF. Data are presented as the mean ± SD (*n* ≥ 3). The solid lines represent nonlinear regression curves used to determine the IC_50_ values.

This selective cytotoxicity towards cancer cells, while preserving normal cells, underscores the promise of Pd-MOF as a potentially safer anticancer agent. The results depicted in Fig. 6A, B underscore the potential of Pd-MOF as a viable chemotherapeutic agent, capable of differentiating between malignant and non-malignant cells. This selectivity not only reinforces its therapeutic potential but also necessitates additional mechanistic investigations to clarify its method of action and to examine its advancement as a targeted anticancer drug. The selectivity profile was initially assessed using WI-38 human lung fibroblasts, a representative non-cancerous cell type commonly utilized in preliminary cytotoxicity screening to evaluate general toxicity toward normal human cells and selectivity against malignant cells. The cytotoxicity of novel compounds has been evaluated using WI-38 fibroblasts in multiple studies^91^. This selectivity toward A549 cells is indicative of anticancer activity. Nevertheless, evaluating selectivity in a more tissue-relevant in vitro model, such as BEAS-2B (a non-tumorigenic lung epithelial cell line), would provide stronger support for this hypothesis. This study is limited by the absence of a normal epithelial lung model. Additional studies using BEAS-2B or similar cell lines are required to confirm the tissue-specific selectivity and translational relevance of Pd-MOF.

#### VEGFR2 kinase inhibitory assay

**Fig. 7:**
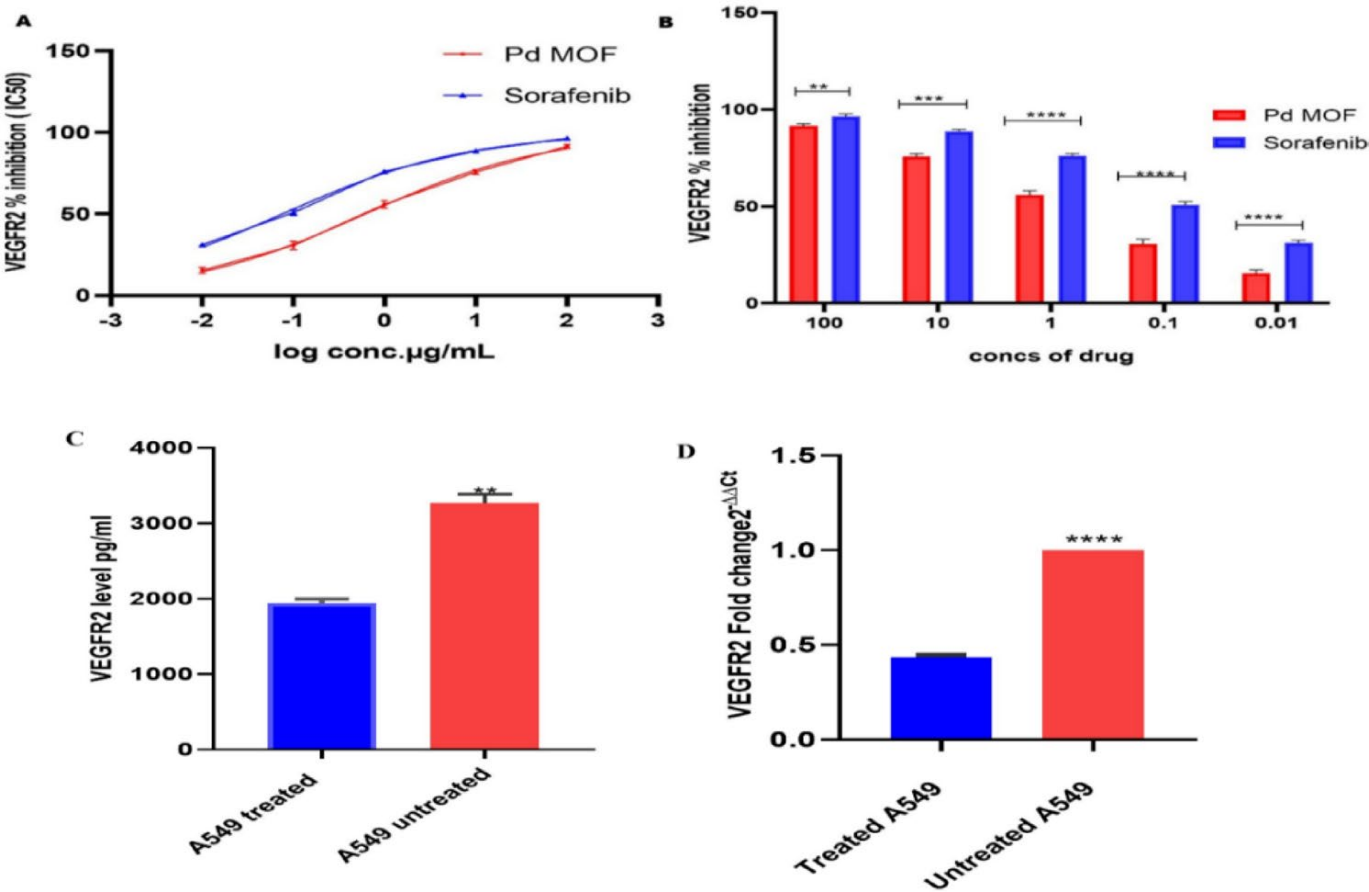
(**A**) VEGFR2 enzyme inhibition expressed as IC_50_ values for Pd-MOF and sorafenib. (**B**) Dose-dependent VEGFR2 inhibition by Pd-MOF compared to sorafenib. Data are presented as the mean ± SD (*n* ≥ 3). Statistically significant differences in percentage inhibition between Pd-MOF and Sorafenib are indicated by asterisks: **P < 0.001, ***P < 0.0001, ****P < 0.00001, as determined by two-way ANOVA. (**C**) The levels of VEGFR2 (pg/mL) showing a significant decrease in A549 cells treated with Pd-MOF compared with untreated cells at *p* = 0.0047, as determined by an unpaired t-test. (**D**) Relative gene expression levels of VEGFR2 showing a highly significant decrease in A549 cells treated with Pd-MOF compared with untreated cells at *p* < 0.00001, as determined by an unpaired t-test.

In the VEGFR2 kinase inhibitory assay, the 1D Pd-MOF compound demonstrated promising inhibitory activity, indicating its potential as a targeted therapeutic agent. This assay was conducted to explore whether the observed cytotoxicity of 1D Pd-MOF could be attributed to interference with VEGFR2 signaling, a key regulator of angiogenesis in tumor progression. As shown in Fig. 7A, B, Pd-MOF exhibited moderate VEGFR2 inhibitory activity, with an IC_50_ value of 0.658 ± 0.023 µg/mL. The percentage of VEGFR2 enzyme inhibition ranged from 15.43% to 91.46% in a dose-dependent manner. For comparison, the reference drug sorafenib, a clinically approved VEGFR2 inhibitor, exhibited an IC_50_ value of 0.079 ± 0.003 µg/mL, with inhibition ranging from 31.13% to 96.43%. Although sorafenib showed higher potency at lower concentrations, the gap in inhibitory efficacy between Sorafenib and Pd-MOF diminished at higher concentrations of Pd-MOF, as illustrated in Fig. 7B. These findings suggest that Pd-MOF moderately inhibits VEGFR2 activity and may exert its anticancer effects, at least in part, by targeting VEGFR2.

These findings were further supported by measuring the intracellular levels of VEGFR2 to evaluate the effect of Pd-MOF on angiogenic signaling. Figure 7C illustrates a significant reduction in the VEGFR2 level in Pd-MOF-treated A549 cells (1940.7 ± 56.2 pg/mL) compared with untreated control cells (3270.2 ± 93.9 pg/mL) (*p* = 0.0047). This significant reduction (approximately 40.6%) indicates that Pd-MOF effectively inhibits VEGFR2-mediated pathways in lung cancer cells. Although Pd-MOF produced a highly significant downregulation of VEGFR2 gene expression in A549 cells compared with the untreated control (*p* < 0.00001; Fig. 7D), its inhibitory potency remained considerably weaker than that of the reference drug, sorafenib. Nevertheless, these findings suggest that Pd-MOF could serve as a promising lead scaffold for further structural optimization to improve its therapeutic efficacy and safety profile.

#### [^3^h] colchicine–tubulin binding assay

**Fig. 8:**
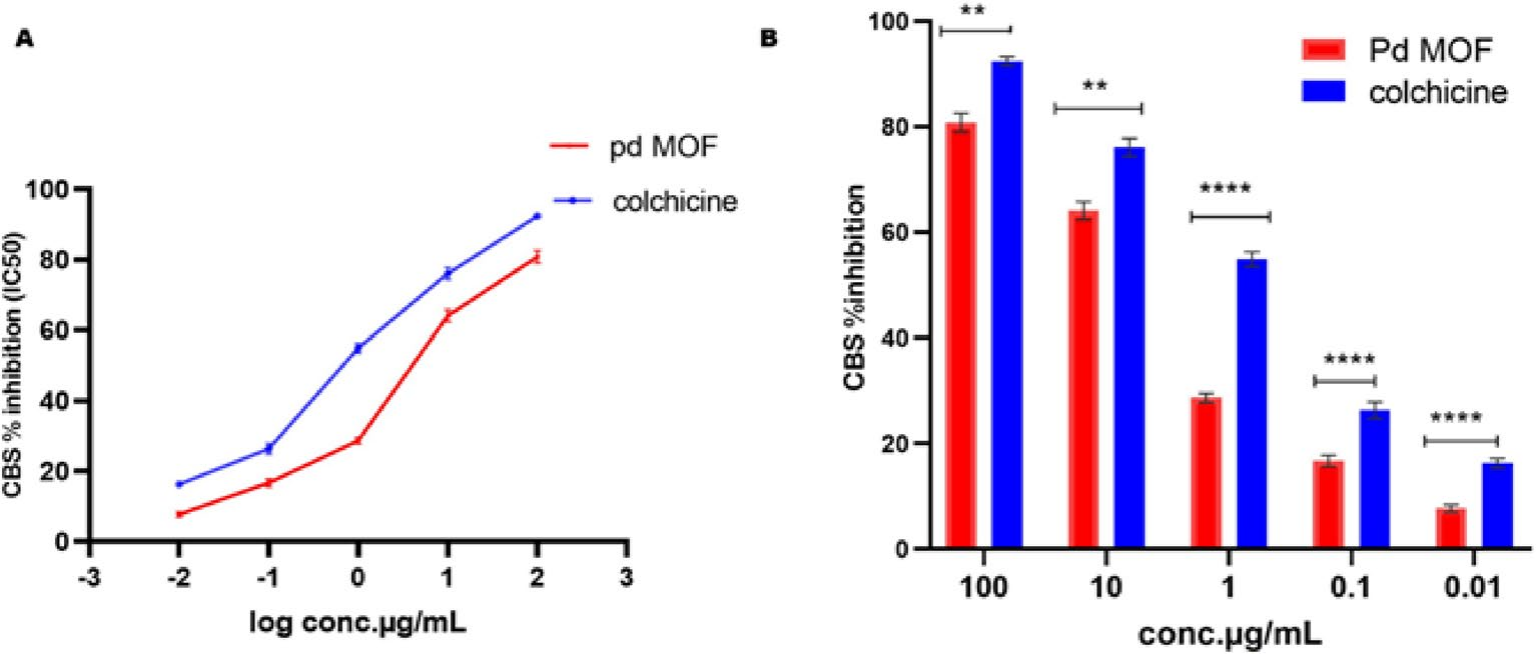
(**A**) Colchicine binding site (CBS) inhibition expressed as IC_50_ values for Pd-MOF and [^3^h]-colchicine. (**B**) Dose-dependent CBS inhibition by Pd-MOF compared to [^3^h]-colchicine. Data are presented as mean ± SD (*n* ≥ 3). Statistically significant differences in percentage inhibition between Pd-MOF and [^3^h]-colchicine are indicated by asterisks: **P < 0.001, ***P < 0.0001, ****P < 0.00001, as determined by two-way ANOVA.

Microtubules, composed of α- and β-tubulin heterodimers, are essential components of the eukaryotic cytoskeleton and play important roles in mitosis, intracellular transport, maintenance of cell shape, and cellular motility. Because of their crucial involvement in cell proliferation, tubulin-binding agents remain important targets in anticancer therapy. To evaluate whether Pd-MOF interacts with the colchicine-binding site (CBS) of tubulin, a competitive [^3^H] colchicine-binding assay was performed using purified tubulin. Colchicine, a well-characterized ligand of the colchicine-binding site, was used as the reference compound. The results demonstrated that Pd-MOF effectively inhibited the binding of [^3^H] colchicine to tubulin in a concentration-dependent manner, with an IC_50_ value of 3.350 ± 0.21 µg/mL, whereas colchicine exhibited an IC_50_ value of 0.710 ± 0.04 µg/mL (Fig. 8A). In competition experiments containing both Pd-MOF and radiolabeled colchicine, Pd-MOF reduced colchicine binding to tubulin, with inhibition ranging from 80.75% to 7.7% over the concentration range of 100 to 0.01 µg/mL. Under the same experimental conditions, colchicine produced inhibition ranging from 92.37% to 16.28% (Fig. 8B). These findings indicate that Pd-MOF interacts with the colchicine-binding site on tubulin and competes with colchicine for binding in vitro. The observed interaction may contribute to the antiproliferative activity of Pd-MOF by interfering with tubulin-associated cellular processes. However, the direct effects of Pd-MOF on tubulin polymerization were not evaluated in the present study and require further investigation using dedicated polymerization assays.

#### Inhibitory activity of NO over-production

Nitric oxide (NO) is one of the most important regulatory and inflammatory mediators, playing a pleiotropic role in numerous physiological and pathological processes, including vasodilation, neurotransmission, and, most notably, host immune defense^92,93^. NO is synthesized through the nitric oxide synthase (NOS)-catalyzed conversion of L-arginine to citrulline^92^. There are three isoforms of NOS: two constitutive forms, endothelial NOS (eNOS) and neuronal NOS (nNOS), and one inducible form (iNOS), which is predominantly expressed in activated macrophages^94^. Excessive NO production, particularly through iNOS in macrophages, has been implicated in inflammation, cytotoxicity, autoimmune disorders, and tumor development^92^. Therefore, the inhibition of excessive NO production is considered a crucial strategy for managing not only inflammatory diseases but also cancer therapy, due to its role in suppressing angiogenesis, tumor invasion, and metastasis^95^.

**Fig. 9:**
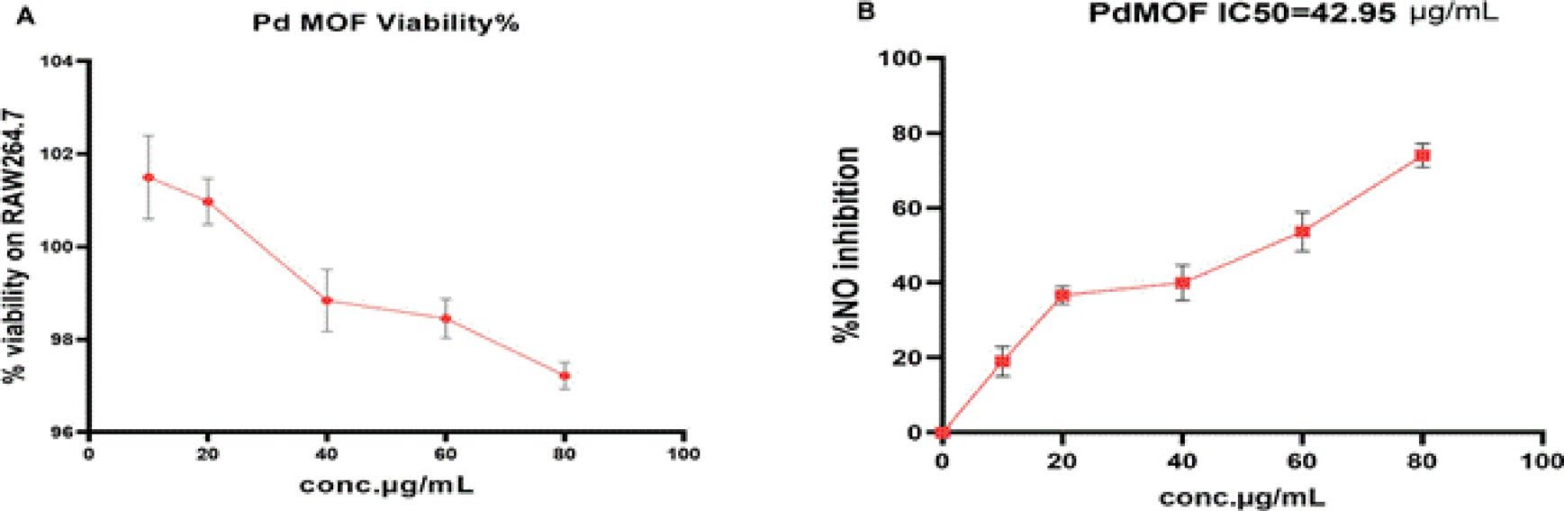
(**A**) Cell viability of RAW 264.7 macrophages following treatment with various concentrations of Pd-MOF. (**B**) Dose-dependent inhibition of nitric oxide (NO) production in LPS-stimulated RAW 264.7 cells treated with Pd-MOF. Data are presented as the mean ± SD (*n* ≥ 3).

Given this context, the ability of Pd-MOF to inhibit NO production was evaluated as an indicator of its potential to suppress iNOS activity and expression. As shown in Fig. 9A, Pd-MOF exhibited no cytotoxic effects on RAW 264.7 macrophage cells, confirming its safety at the tested concentrations. Figure 9B illustrates that Pd-MOF significantly inhibited NO release in a dose-dependent manner, achieving 19.01% to 74.03% inhibition, with an IC_50_ value of 42.95 µg/mL. This activity was compared with the reference anti-inflammatory agent quercetin, which demonstrated an IC_50_ of 9.06 µg/mL.

These results suggest that Pd-MOF is a promising anti-inflammatory candidate capable of reducing LPS-induced NO overproduction without compromising cell viability. Its dose-dependent inhibition of NO release suggests a potential mechanism of action through iNOS suppression. Consequently, Pd-MOF may serve as an effective agent for the management of inflammation-related disorders, including cancer, through modulation of macrophage-mediated NO production. Although the current anti-inflammatory evaluation focused on NO inhibition as a key inflammatory mediator, further investigations targeting downstream signaling pathways and other pro-inflammatory cytokines (e.g., TNF-α, IL-6) are warranted in future studies to fully elucidate the underlying molecular mechanisms.

#### The apoptotic cell death by Pd-MOF

##### Cell cycle analysis

Signal transduction and proliferative pathways play a critical role in cancer progression and metastasis by modulating the activation and function of proteins associated with cell cycle regulation^96^. Genomic instability is a hallmark of cancer and plays a central role in both tumor initiation and progression. In healthy cells, genomic integrity is maintained by tightly regulated checkpoints at various stages of the cell cycle, which ensure DNA damage is either repaired or leads to cell cycle arrest or apoptosis if irreparable. However, in cancer cells, one or more of these checkpoints are often disrupted, allowing for the accumulation of mutations and uncontrolled proliferation. This genomic heterogeneity provides a selective advantage under environmental stressors such as hypoxia, therapeutic interventions, and immune surveillance. Understanding the molecular mechanisms governing these checkpoints in tumor cells offers promising opportunities for targeted therapeutic strategies^96^.

**Fig. 10:**
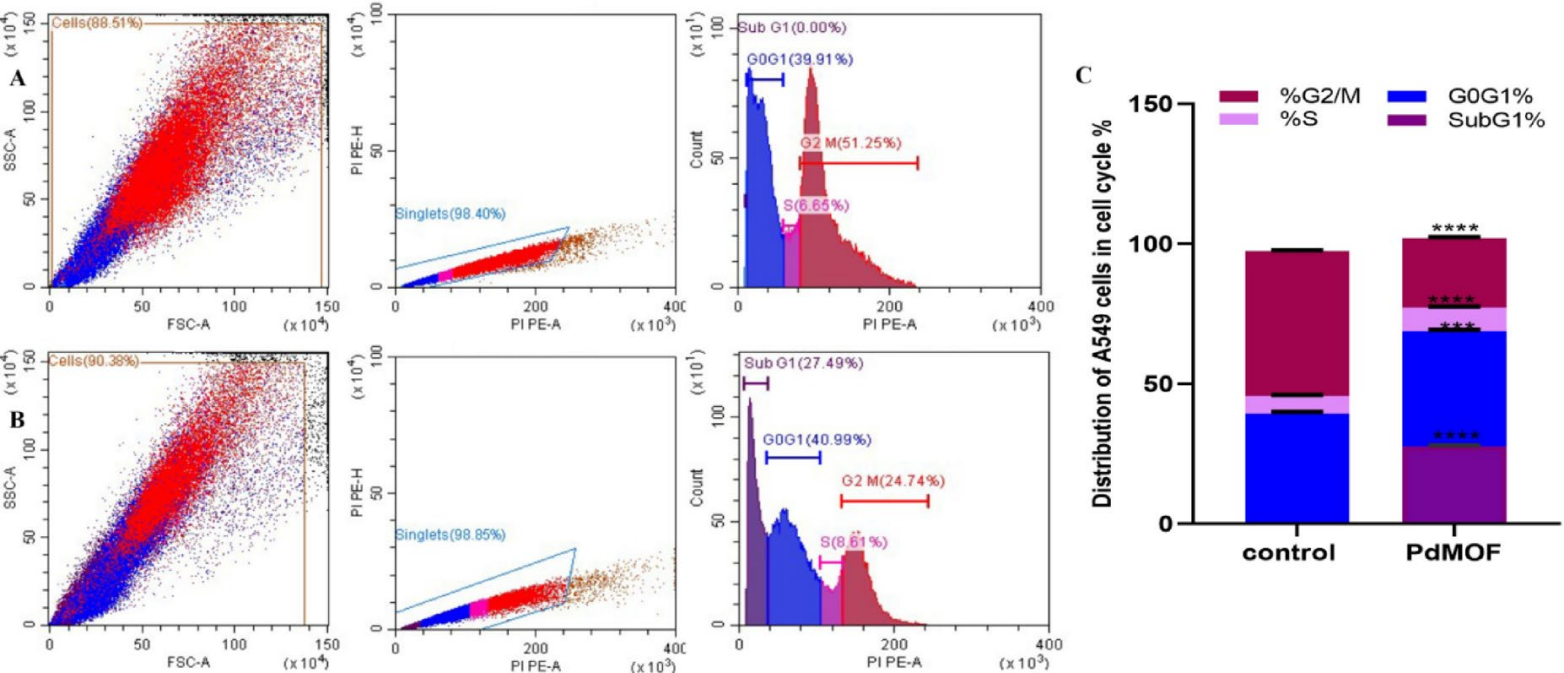
(**A**) Representative histogram of the cell cycle distribution in untreated A549 control cells, analyzed by propidium iodide (PI) staining. (**B**) Representative histogram of A549 cells after 48 h of treatment with Pd-MOF at its IC_50_ concentration. Treatment induced a marked increase in the sub-G1 population from 0.00% (control) to 27.49% (treated), accompanied by a corresponding reduction in the G2/M phase population, indicating apoptosis. (**C**) Quantitative representation of the percentage of cells in each phase of the cell cycle. Data are presented as the mean ± SD from three independent experiments. Statistically significant differences between treated and control groups are indicated by asterisks: ***P < 0.0001 and **P < 0.00001, as determined by two-way ANOVA.

To investigate whether Pd-MOF exerts antiproliferative effects through modulation of cell cycle progression, flow cytometric analysis was conducted on A549 lung cancer cells treated with Pd-MOF at its IC_50_ concentration for 48 h. The results demonstrated that Pd-MOF significantly altered cell cycle distribution compared with untreated controls. As shown in Fig. 10, these results help elucidate the mechanism underpinning its anti-proliferative activity. In contrast to the untreated control cells, the cell cycle profile was significantly altered following Pd-MOF treatment, as demonstrated by the data. The most significant finding was a substantial and abrupt rise in the sub-G1 population, a recognized indicator of apoptotic cell death characterized by DNA fragmentation, which increased from 0.00% in the control group to 27.49% in the Pd-MOF-treated group. A significant decrease in the proportion of cells in the G2/M phase was observed, decreasing from 51.25% (control) to 24.74% after treatment, indicating a pronounced inhibition of mitotic development. In contrast, the percentages of cells in the G0/G1 and S phases showed only moderate changes. Collectively, these findings reveal a substantial accumulation of cells in the sub-G1 fraction, accompanied by a reduction in the G2/M phase, indicating that the cytotoxic efficacy of Pd-MOF is primarily mediated through apoptosis rather than arrest at a specific cell cycle checkpoint. This mechanism highlights the therapeutic potential of Pd-MOF as a candidate for further development in anticancer strategies targeting cell cycle regulation.

#### Annexin V apoptosis assay

**Fig. 11:**
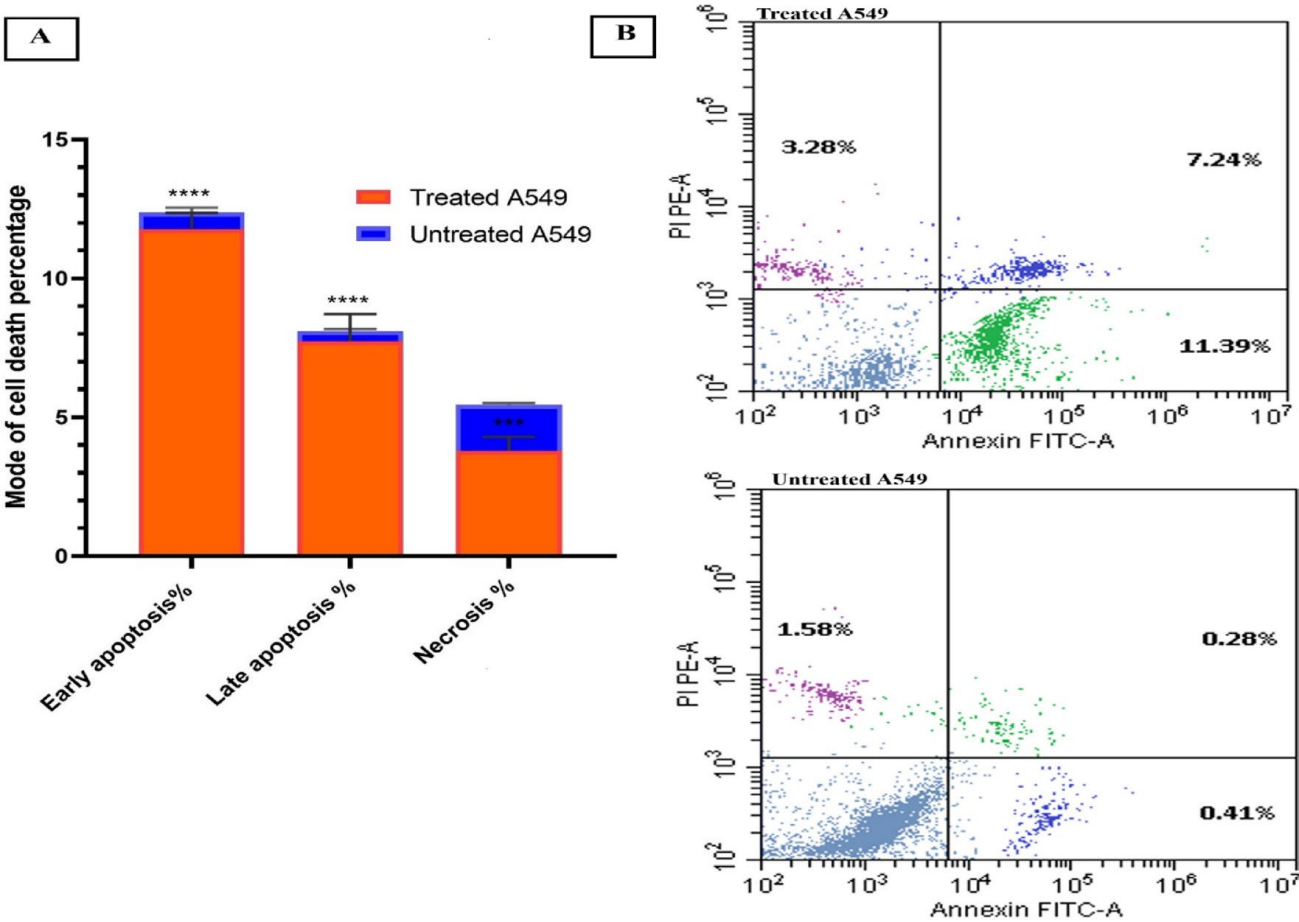
(**A**) Quantitative analysis of the cell populations in untreated and Pd-MOF-treated A549 cells. Data are presented as the mean ± SD from three independent experiments. Statistically significant differences between treated and control groups are indicated by asterisks: ***P < 0.0001 and **P < 0.00001, as determined by two-way ANOVA. (**B**) Representative Annexin V-FITC/PI dot plots showing the percentages of viable, early apoptotic, late apoptotic, and necrotic A549 cells following treatment with Pd-MOF compared with untreated control cells.

To further elucidate the mechanism of cell death, whether apoptotic or necrotic, induced by Pd-MOF in A549 lung cancer cells, Annexin V-FITC/PI dual staining followed by flow cytometric analysis was performed (Fig. 11). The results revealed that Pd-MOF treatment significantly increased both early and late apoptotic cell populations, with a total apoptosis rate of 9.77% compared with only 0.48% in untreated control cells. This marked increase suggests that Pd-MOF acts as a potent inducer of apoptosis. Additionally, a smaller proportion of cells (3.8%) underwent necrotic (non-programmed) cell death following treatment, indicating that apoptosis is the predominant mode of cell death triggered by Pd-MOF.

#### Real-time-polymerase chain reaction for the selected genes

The novel compound Pd-MOF underwent molecular investigation on the A549 lung cancer cell line, demonstrating high cytotoxic efficacy and selectivity. As detailed in previous sections, Pd-MOF exhibited a potent antiproliferative effect with the lowest IC_50_ value (78.21 ± 0.41 µg/mL) against A549 cells, while showing comparatively lower toxicity toward the normal WI-38 fibroblast cell line. To further explore the underlying molecular mechanism of its anticancer activity, particularly the pathways involved in apoptosis and cell cycle regulation, real-time PCR analysis was performed. A549 cells were treated with the IC_50_ concentration of Pd-MOF for 48 h at 37 °C, after which total RNA was extracted from both treated and untreated cells for gene expression analysis.

**Fig. 12:**
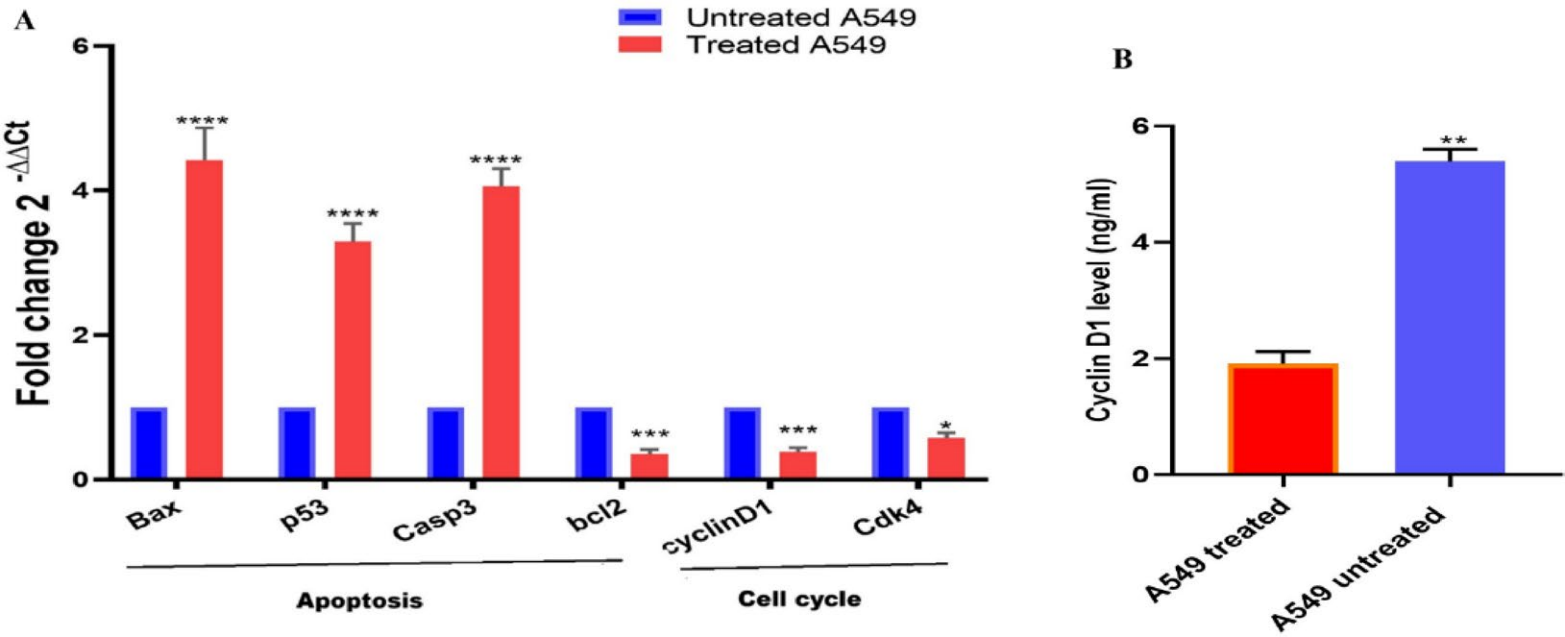
(**A**) Relative gene expression levels of pro-apoptotic markers (*BAX*,* P53*, and *Caspase-3*), anti-apoptotic marker (*Bcl-2*), and cell cycle regulators (*Cyclin D1* and *CDK4*) in A549 lung cancer cells treated with Pd-MOF compared to untreated control cells, as measured by real-time PCR. Data are presented as the mean ± SD from three independent experiments. Statistically significant differences between treated and control groups are indicated by asterisks: *P < 0.001, ***P < 0.0001, ****P < 0.00001, as determined by two-way ANOVA. (**B**) The level of Cyclin D1 (ng/mL) shows a significant decrease in A549 cells treated with Pd-MOF when compared with untreated cells at *p* = 0.0036 as determined by an unpaired t-test.

The results revealed a significant upregulation of pro-apoptotic genes in Pd-MOF-treated cells: **BAX** showed a 4.42-fold increase, **caspase-3** was upregulated by 4.06-fold, and the tumor suppressor gene **p53** exhibited a 3.29-fold increase in expression. In contrast, a marked downregulation was observed in the expression of anti-apoptotic and cell cycle regulatory genes, respectively: **Bcl-2** expression decreased to 0.35-fold, **cyclin D1** to 0.39-fold, and **CDK4** to 0.58-fold relative to control as observed in Fig. 12A.

These findings indicate that Pd-MOF effectively promotes apoptosis by enhancing pro-apoptotic signals while simultaneously suppressing survival and proliferation pathways. The concurrent upregulation of BAX, p53, and caspase-3 and the downregulation of Bcl-2.

To further elucidate the mechanisms underlying cell cycle arrest, the expression profiles of key regulatory proteins, specifically cyclin D1 and CDK4, were examined. Quantitative analysis revealed that Pd-MOF treatment resulted in statistically significant downregulation of both cyclin D1 (*p* < 0.0001) and CDK4 (*p* < 0.001) expression (Fig. 12A). These findings were corroborated by protein concentration assays, which demonstrated a significant reduction in cyclin D1 levels (*p* = 0.0036) (Fig. 12B). The depletion of these critical G1 regulatory components effectively inhibits cell cycle progression at the G0/G1 checkpoint. The induction of cellular stress by Pd-MOF and subsequent arrest of the cell cycle appears to activate apoptotic signaling pathways, as evidenced by DNA fragmentation and a corresponding increase in the sub-G1 population^97^. The significant attenuation of cyclin D1 and CDK4 expression suggests that Pd-MOF facilitates programmed cell death and promotes sub-G1 phase accumulation. Consequently, the downregulation of cyclin D1 may function as a primary upstream mediator of the apoptotic cascade, underscoring the potential of Pd-MOF as a novel therapeutic candidate for lung carcinoma.

#### Dual acridine orange/ethidium bromide (AO/EB) fluorescent staining double staining assay

**Fig. 13:**
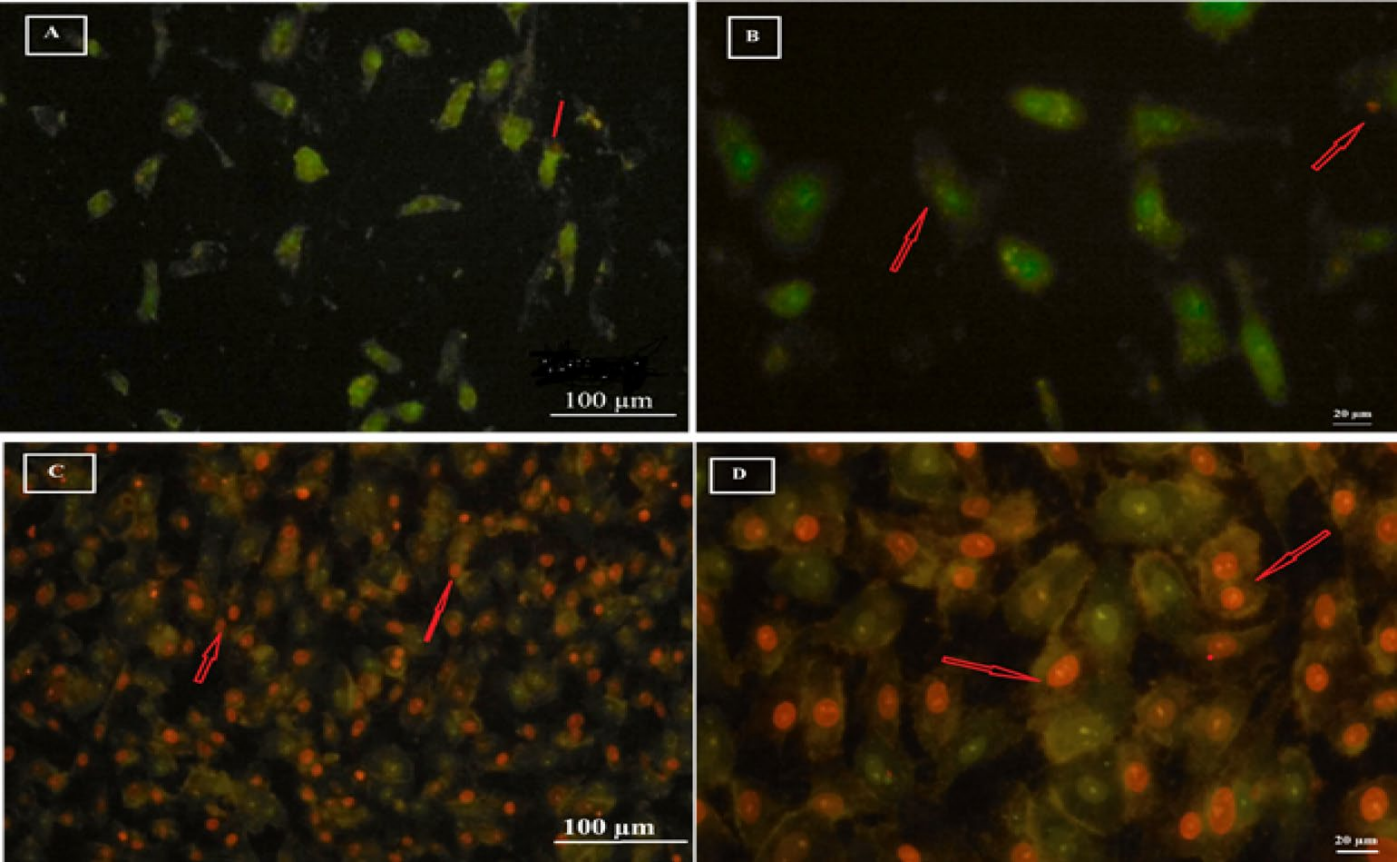
Morphological and nuclear alterations in A549 cells visualized before and after treatment with Pd-MOF, respectively. Cells were stained with AO/EB and observed under a fluorescence microscope. Untreated control cells show intact nuclei with minimal signs of early apoptosis (red arrows) at magnifications of 10× (**A**) and 40× (**B**). Pd-MOF–treated cells exhibit distinct features of apoptosis, including early and late apoptotic bodies (red arrows) at magnifications of 10× (**C**) and 40× (**D**).

To further characterize the morphological changes induced by Pd-MOF treatment in A549 cells, dual AO/EB fluorescent staining was performed as described by Kasibhatla et al.^98^. After 48 h of treatment, untreated control A549 cells exhibited a well-organized morphology, with round, intact nuclei showing green fluorescence, indicative of healthy cells. In contrast, Pd-MOF-treated A549 cells displayed bright orange fluorescence, characteristic of early apoptotic cells, as well as red fluorescence, indicative of late apoptotic cells. These observations suggest that Pd-MOF treatment effectively induces apoptosis in A549 cells, confirming its potential as an apoptosis-inducing agent (Fig. 13).

#### Structure–activity relationship (SAR)

The observed biological activity of the synthesized 1D Pd-MOF can be rationalized based on its structural and electronic characteristics. The square-planar Pd(II) coordination environment, which shares important coordination and structural features with Pt(II)-based anticancer agents, provides a favorable metal center for interactions with biological macromolecules^99^. In addition, the rigid pyrazine ligand acts as an N, N-donor bridge, generating a one-dimensional polymeric framework that stabilizes multiple Pd(II) centers while preserving their accessibility for interaction with biological targets^99,100^. Furthermore, the reduced HOMO–LUMO energy gap obtained from the DFT calculations, together with the molecular electrostatic potential (MEP) and natural bond orbital (NBO) analyses, indicates enhanced electronic reactivity and a favorable distribution of electron-rich and electron-deficient regions that may facilitate intermolecular interactions with biomolecular targets^101^. The combination of the polymeric architecture, accessible Pd(II) coordination sites, and favorable electronic properties provides a reasonable explanation for the experimentally observed multitarget biological activity, including VEGFR2 inhibition, interaction with the colchicine-binding site of tubulin, apoptosis induction, and anti-inflammatory activity^102^.

### Molecular docking investigation

**Fig. 14:**
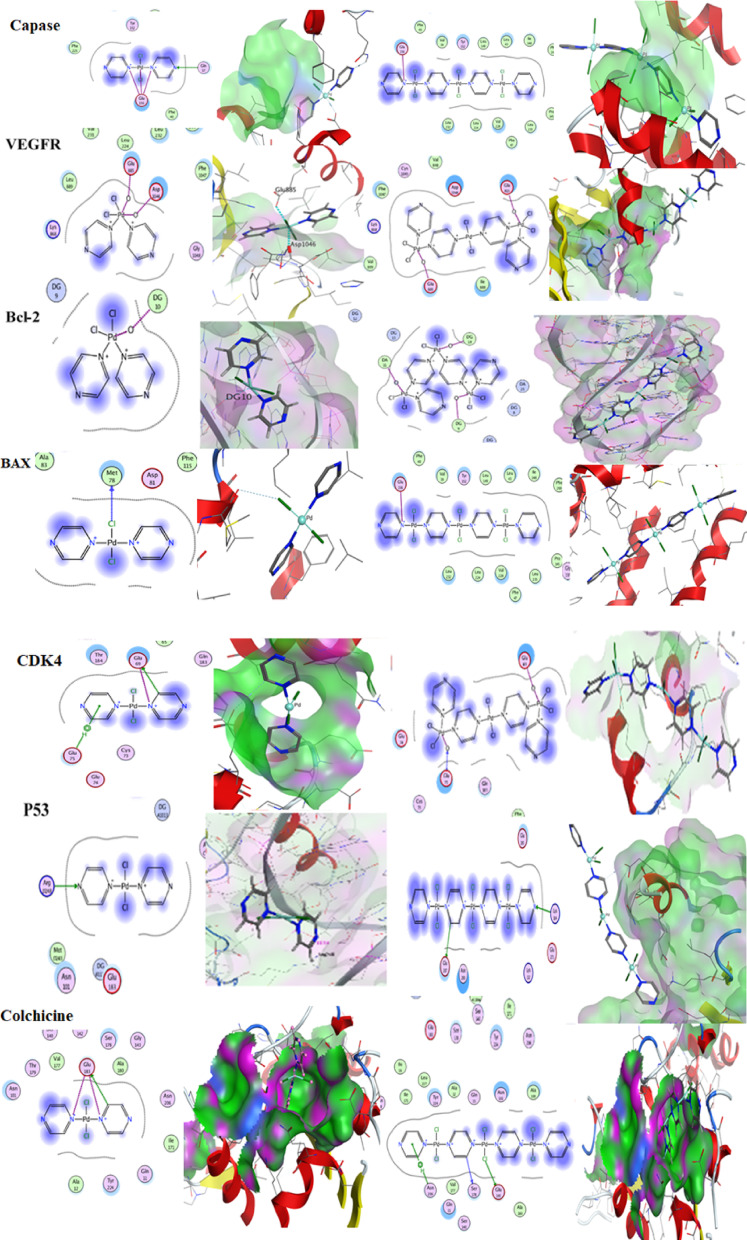
2D and 3D docking results of monomeric and polymeric forms of Pd based pyrazin with VEGFR2 kinase, cleaved Caspase 3, Bcl-2, BAX, CDK4, P53 and Colchicine Site of Tubulin using (Code: 3BE2, 7P16, 6ZX7, 5W63, 2W9Z, 1TUP and 4O2B, respectively).

The enzymes discovered during apoptosis investigation for cell cycle apoptosis were subjected to molecular docking analysis. The docking is based on palladium-pyrazine chains, which include one and three palladium nuclei. The scoring energy values indicated the binding affinity of the complex with the selected proteins for each enzyme. All proteins demonstrated substantial binding affinity to the specified proteins, as indicated in Table S3, based on the energy scoring values. The binding affinity of the polymeric form exceeds that of the monomeric structure. This may pertain to the poly nucleus of palladium ions that engage in contacts with the active amino acids via metal contact interaction, as seen in Fig. 14. Consequently, it was anticipated that elongating the polymeric chain would augment the active metal sites, thereby enhancing the biological interactions with each protein, which supports the results in the MTT research section. Hydrogen bonding related to the backbone donor and the sidechain donor interaction types was also identified. Ultimately, it was determined that the types of interactions enhance the stability of the protein complex, aiding in the suppression of cancer cell proliferation, cell cycle advancement, and the promotion of apoptosis.

To validate the molecular docking protocol, the co-crystallized ligand associated with each target protein was re-docked into its original binding site using the same docking parameters. The obtained root mean square deviation (RMSD) values ranged from 0.72 to 2.90 Å, which fall within the generally accepted validation criterion (RMSD < 3.0 Å), indicating satisfactory agreement between the experimental and predicted binding modes and confirming the reliability of the docking protocol. In addition, 5-fluorouracil (5-FU) was included as a standard anticancer drug to provide a reference for comparison of the binding affinities of the investigated palladium compounds. The docking scores of the standard drug and validation linker are summarized in Table S3, while the corresponding binding modes are presented in Figure S4.

## Conclusion

Establishing a therapeutic approach for managing cancer using **VEGFR2** kinase inhibition and additionally uncontrolled inflammation predisposes to pleiotropic effects leading to cancer development and promoting all stages of tumorigenesis. A 1D MOF was prepared and its structure based on poly palladium and pyrazine. DFT study confirmed the suggested structure with good agreement between the theoretical and experimental spectral results. The synthesized Pd-MOF demonstrated potent antiproliferative activity against A549 lung cancer cells (IC_50_ = 78.21 ± 0.41 µg/mL) while exhibiting moderate selectivity toward normal WI-38 cells compared with the reference drug 5-fluorouracil. Pd-MOF exhibited moderate VEGFR2 inhibitory activity compared with sorafenib and significantly reduced both VEGFR2 gene expression and protein levels in treated A549 cells relative to untreated controls. Our findings also indicate that Pd-MOF competes with colchicine for binding to tubulin, suggesting interaction with the colchicine-binding site in vitro. This interaction may contribute to the antiproliferative activity of Pd-MOF through interference with tubulin-associated cellular processes (IC_50_ = 3.350 ± 0.21 µg/mL), supporting tubulin as a potential molecular target. Pd-MOF markedly upregulated BAX, p53, and caspase-3 expression while downregulating CDK4, cyclin D1, and BCL-2 expression in A549 cells, suggesting growth inhibition and activation of apoptotic signaling. These findings support the conclusion that the 1D Pd-MOF exerts anticancer activity by inducing programmed cell death through apoptosis. Flow cytometric analysis demonstrated substantial accumulation of cells in the sub-G1 fraction, accompanied by a reduction in the G2/M phase, indicating that the cytotoxic effects of Pd-MOF are primarily mediated through apoptosis rather than arrest at a specific cell cycle checkpoint. The anti-inflammatory activity of Pd-MOF was demonstrated by its inhibition of nitric oxide production in LPS-stimulated RAW264.7 macrophages. Molecular docking demonstrated favorable binding of the 1D Pd-MOF to the investigated protein targets, supporting the experimentally observed biological activities. Although the present study demonstrates promising in vitro activity, further in vivo investigations are warranted to validate these findings in more complex biological systems.

## Supplementary Information

Below is the link to the electronic supplementary material.

Supplementary Material 1

## Data Availability

Data is available on request.
