# Supplementary material for: One-dimensional palladium MOF as VEGFR2 and colchicine binding inhibitors with potential anticancer and anti-inflammatory activities: synthesis and molecular investigation
Source: Sci Rep. 2026 Jul 30;16:23653. doi: 10.1038/s41598-026-63591-z (PMC13424319; doi:10.1038/s41598-026-63591-z)
Supplement: Supplementary file 1 — Supplementary Material 1 [file 41598_2026_63591_MOESM1_ESM.docx]

**One-dimensional Palladium MOF as VEGFR2& Colchicine binding inhibitors with potential anticancer and anti-inflammatory activities: Synthesis and molecular investigation**

**Heba K. Abdelhakim^1*^, Safaa S. Hassan^2*^ and Khaled M. Ismail^2*^**

**^1^Biochemistry Division, Chemistry Department, Faculty of Science, Cairo University, Egypt**

**^2^Chemistry Department, Faculty of Science, Cairo University, Giza 12613, Egypt.**

**Corresponding author:** [**kismail@sci.cu.edu.eg**](mailto:kismail@sci.cu.edu.eg)**,** [**Hebak@sci.cu.edu.eg**](mailto:Hebak@sci.cu.edu.eg)**, hsafaa@sci.cu.edu.eg**

**Biological evaluation**

***Antiproliferative activity by MTT assay: -***

Lung cancer (A549) and normal (WI-38) cell lines were obtained from Nawah Scientific (Cairo, Egypt). According to ATCC recommendations, cells were cultured in Dulbecco’s Modified Eagle Medium (DMEM) supplemented with 10% fetal bovine serum (Gibco, Life Technologies Inc., UK) and 1% penicillin-streptomycin (Gibco). Cells were preserved and grown in a moist environment with 5% CO_2_ at 37°C.

Cell viability was assessed using the MTT assay, a colorimetric method based on the enzymatic reduction of MTT [3-(4,5-dimethylthiazol-2-yl)-2,5-diphenyl tetrazolium bromide] to purple formazan crystals by mitochondrial succinate dehydrogenase in metabolically active cells. The quantity of formazan produced, directly proportional to the number of viable cells, was measured spectrophotometrically.

Once seeded, the cells were incubated at 37°C for 24 hours to allow attachment and growth. Subsequently, media containing various concentrations of 1D Pd-MOF (1000, 500, 250, 125, 62.5, and 31.25 µg/mL) were added. After 48 hours of treatment, the media were removed, and 100 µL of MTT solution (0.5 mg/mL) was added to each well. The plates were incubated for 4 hours, after which 100 µL of DMSO (Sigma-Aldrich) was added to dissolve the formazan crystals. The plates were gently shaken for 15 minutes, and absorbance was measured at 492 nm using a microplate reader.

Relative cell viability was calculated by comparing absorbance values between treated and untreated cells. The IC₅₀ value (the concentration that inhibits 50% of cell viability) was determined after 48 hours of incubation. The IC₅₀ was also evaluated for the non-cancerous WI-38 cell line^59^. In addition, the selectivity index has been calculated for Pd-MOF & standard drug 5-flourouracil.

***2.2.4 Inhibitory activity of NO over-production.***

After confirming the cytocompatibility of the Pd-MOF compound in RAW264.7 macrophage cells, its anti-inflammatory potential was further investigated using an LPS-induced inflammation model. Lipopolysaccharides (LPS), which are structural components of many pathogenic bacteria, fungi, and viruses, were used to mimic an inflammatory environment. LPS activates inflammatory pathways, such as the MAPK and NF-κB signaling cascades, through Toll-like receptors expressed on macrophages.

Common inflammatory markers assessed in such assays include nitric oxide (NO) production, inducible nitric oxide synthase (iNOS) protein expression, and interleukin-6 (IL-6) secretion. The ability of test compounds to suppress these LPS-induced inflammatory responses is indicative of their anti-inflammatory efficacy.

RAW264.7 cells were seeded into 96-well plates and incubated for 24 hours. Inflammation was then induced by treating cells with 1 µg/mL of LPS (LPS group), while untreated cells received fresh media (control group). The test compound was co-administered with LPS at five different concentrations (LPS + Drug groups). Quercetin served as a positive control for anti-inflammatory activity.

To quantify NO production, equal volumes of the cell culture supernatant and Griess reagent were mixed and incubated in the dark at room temperature for 10 minutes. The absorbance was measured at 540 nm using an ELISA plate reader, reflecting the nitrite concentration as an indicator of NO release and determined by measuring as % inhibition for NO production at each concentration ^44^.

***Real-time-polymerase chain reaction for the selected genes.***

To evaluate the apoptotic potential of 1D Pd-MOF, the expression of pro-apoptotic genes (*p53*, *Bax*, and *Caspase-3*), the anti-apoptotic gene (*Bcl-2*), and cell cycle-related genes (*Cyclin D1* and *CDK4*) was analyzed in treated A549 cells using quantitative real-time PCR (qPCR)^66^.

Total RNA was extracted from both untreated (control) and 1D Pd-MOF–treated A549 cells (treated with the compound at its IC₅₀ concentration for 48 h) using the Qiagen RNA extraction kit, following the manufacturer’s protocol. RNA concentration and purity were assessed spectrophotometrically at 260 nm. First-strand cDNA synthesis and subsequent qPCR were performed using the SensiFAST™ SYBR Hi-ROX One-Step Kit (Bioline, UK; Catalog No. PI-50217 V), according to the manufacturer's instructions.

Primers for the target genes (*p53*, *Bax*, *Caspase-3*, *Bcl-2*, *Cyclin D1*, and *CDK4*) and the housekeeping gene (*GAPDH*) were designed to meet optimal parameters, including melting temperature (60–65°C), GC content (40–60%), and amplicon length (90–200 bp). Primer sequences are listed in **Table S1**.

The qPCR reaction mixture had a total volume of 20 μL, which included 4 μL of RNA template. The thermal cycling conditions were as follows: reverse transcription at 45°C for 10 minutes, initial denaturation at 95°C for 10 minutes, followed by 40 cycles at 95°C for 15 seconds (denaturation), 60°C for 30 seconds (annealing), and 72°C for 30 seconds (extension). No-template controls (NTCs): NTCs were included in every qPCR experiment and showed no detectable amplification, confirming the absence of contamination.Additionally no-reverse transcriptase (No-RT) controls: No-RT controls were included to exclude genomic DNA contamination and showed no amplification. Reactions were performed on a StepOne Real-Time PCR System (Applied Biosystems, Foster City, USA).

Gene expression levels were calculated using the ΔΔCt method (2^–ΔΔCt^), with *GAPDH* as the internal control. Results are reported as relative quantification (RQ) values based on three independent experiments.

∆∆Ct = [(Ct_target; sample_) − (Ct_ref; sample_)] − [(Ct _target; control_) − Ct_ref; control_)]

where Ct _target; control_ = Ct value of gene of interest in control DNA.

Ct_ref; control_ = Ct value of reference gene in control DNA.

Ct_target; sample_ = Ct value of gene of interest in the tested sample.

Ct_ref; sample_ = Ct value of reference gene in a tested sample.

The PCR amplification products were separated by electrophoresis on a 1.7% agarose gel to confirm the specificity and expected size of the amplified fragments.

**Table S1. Primer sequences used in the RT-PCR assay for the target genes (*Caspase-3, P53, Bax, Bcl-2, Cyclin D1,* and *CDK4*) and the housekeeping gene (*GAPDH*).**

| **Genes** | **Primer Sequences** |
| --- | --- |
| **Caspase3**  **P53**  **Bax**  **Bcl2**  **CyclinD1**  **Cdk4**  **VEGFR2**  **GAPDH** | F 5’- GGAAGCGAATCAATGGACTCTGG-3’,  R 5'- GCATCGACATCTGTACCAGACC-3'.  F 5’- CCTCAGCATCTTATCCGAGTGG -3’  R 5'- TGGATGGTGGTACAGTCAGAGC-3'  F 5’-TCAGGATGCGTCCACCAAGAAG -3’,  R 5'-TGTGTCCACGGCGGCAATCATC -3'.  F 5’- ATCGCCCTGTGGATGACTGAGT-3’,  R 5'- GCCAGGAGAAATCAAACAGAGGC-3'.  F 5’- AGACCTGCGCGCCCTCGGTG-3’,  R 5'- GTAGTAGGACAGGAAGTTGTTC-3'  F 5’- CCATCAGCACAGTTCGTGAGGT-3’,  R 5'- TCAGTTCGGGATGTGGCACAGA-3'  F 5'- GGC ATC ACG GAA GTG TAT CC -3'  R 5'- CGT TCT TTT TAA CCT GCT TCC AT -3'  F 5’- CACCATTGGCAATGAGCGGTTC-3’  R 5’- AGGTCTTTGCGGATGTCCACGT-3’ |


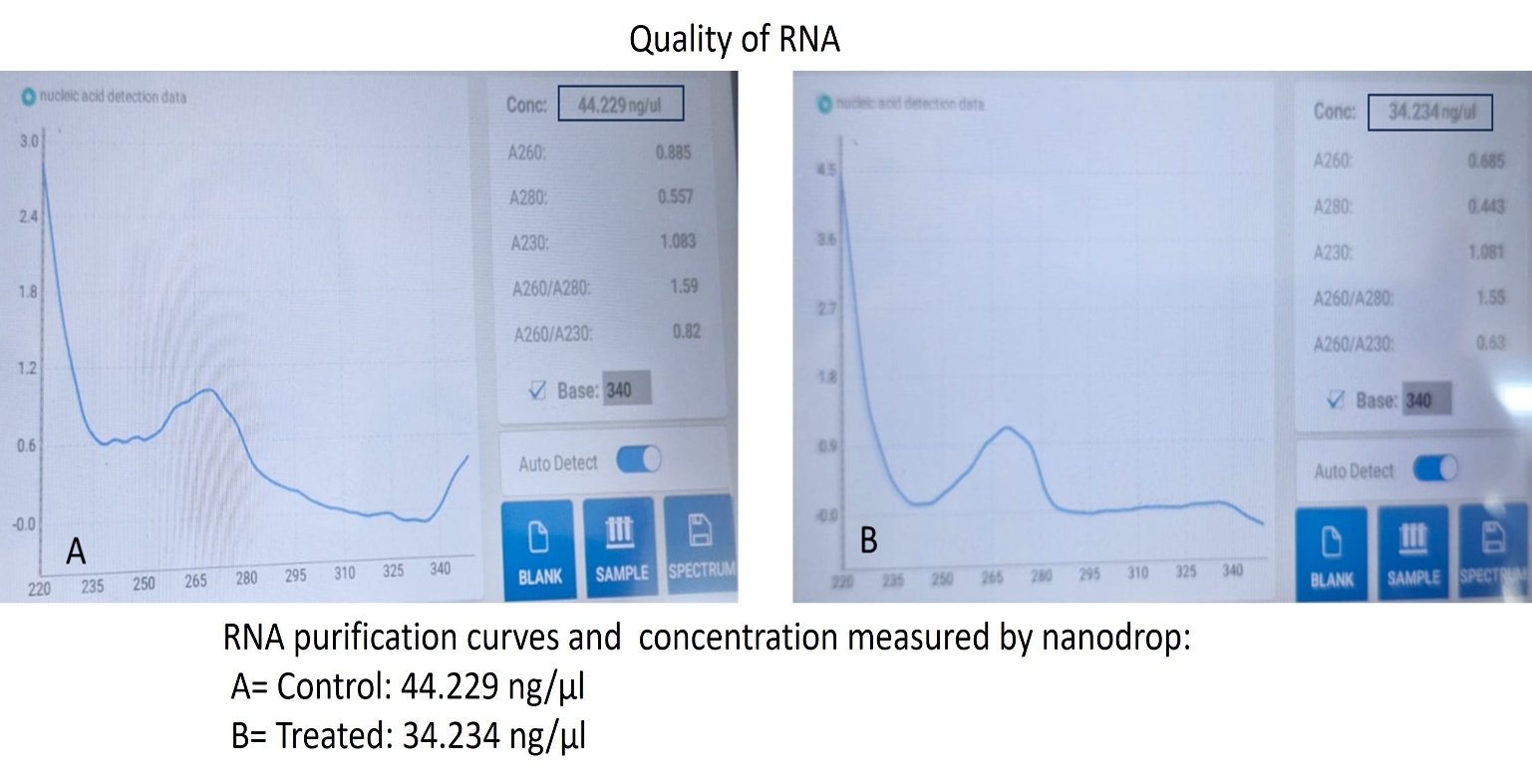

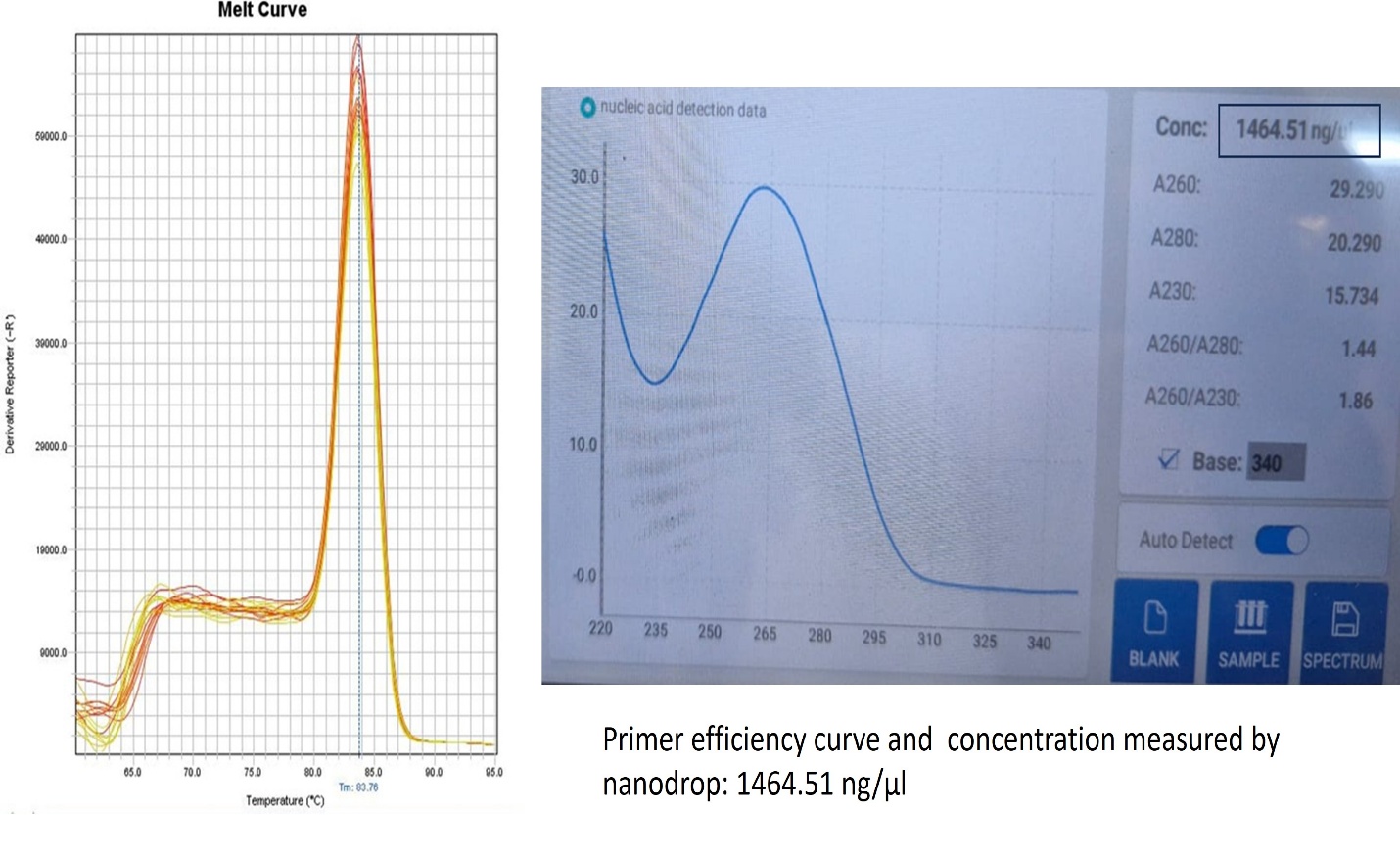
**Figure S1.** primer efficiency, melt-curve analysis

**Figure S2.** RNA quality


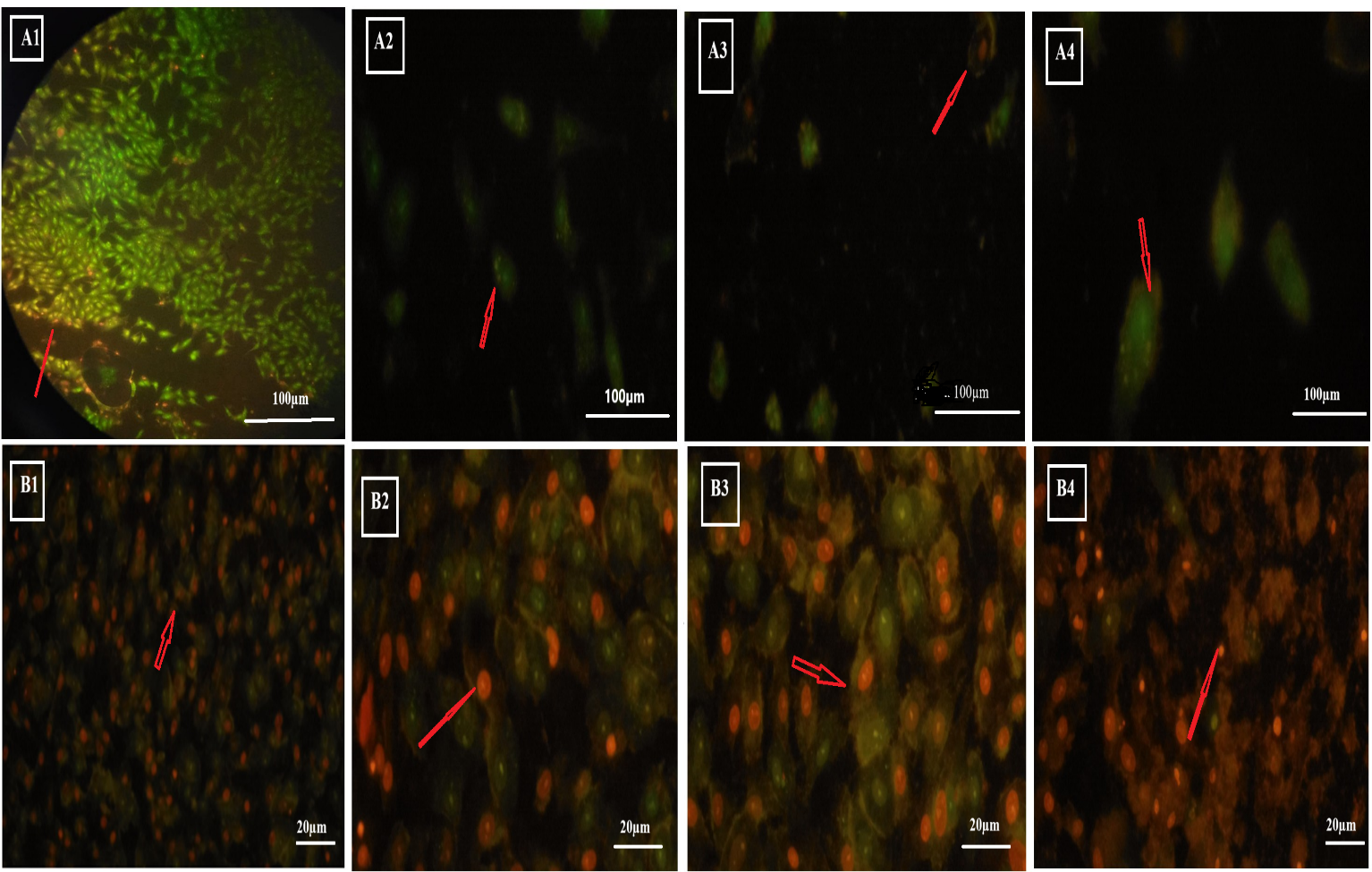
**Figure S3. Morphological and nuclear alterations in A549 cells visualized before and after treatment with Pd-MOF, respectively for four experiments symbolized by A_n_ &B_n_ since (n =1-4) independent experiments. Cells were stained with AO/EB and observed under a fluorescence microscope. (Upper panel from A_1_ to A_4_) Control (untreated) cells show intact nuclei with minimal signs of early apoptosis indicated by red arrows with magnification of 10x. (Lower Panel from B_1_ to B_4_ ) Pd-MOF–treated cells exhibit distinct features of apoptosis, including early and late apoptotic bodies, indicated by red arrows with magnification 40x.**


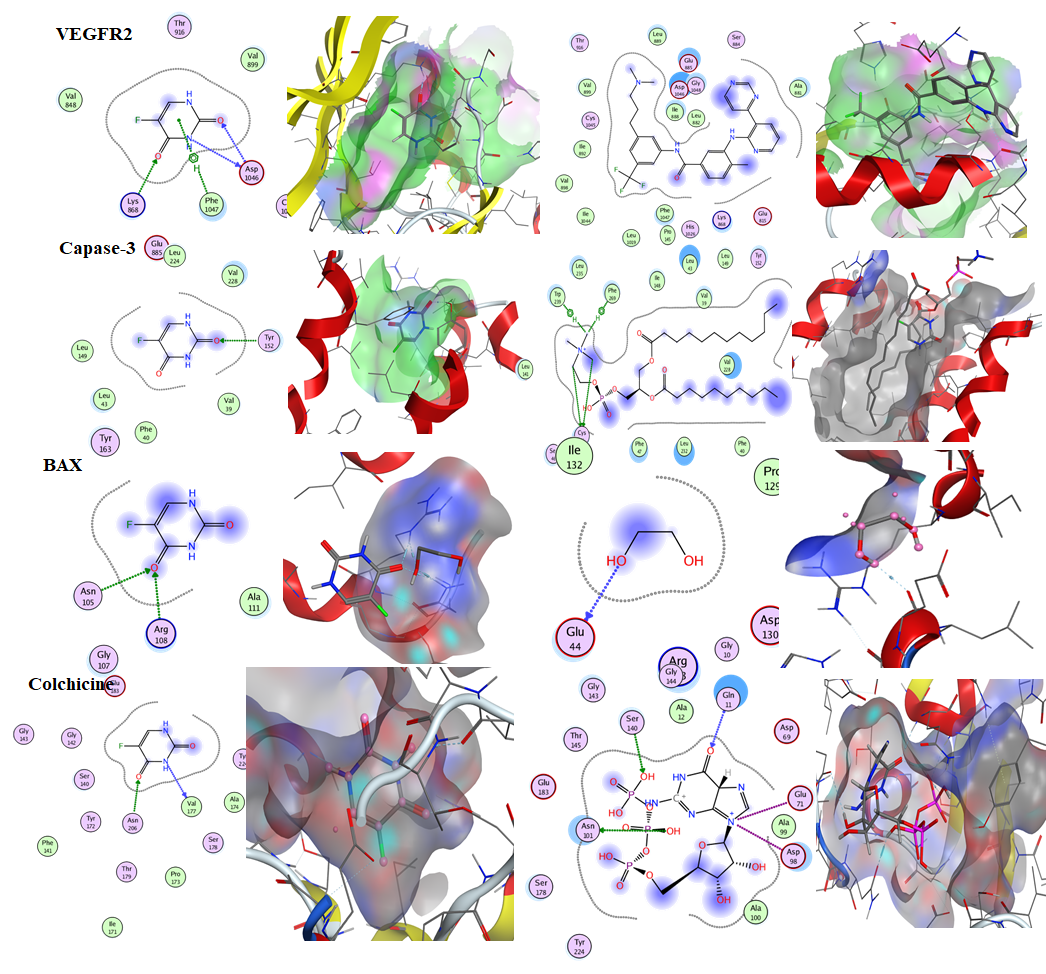


**Figure S4. 2D and 3D docking results of 5-FU standard drug and the co-crystallized ligand for each protein, respectively.**

**2.4. Computational Study:**

Geometry optimization of the pyrazine ligand and the 1D Pd-MOF complex was performed using Density Functional Theory (DFT) with the B3LYP functional. For pyrazine, the 6-311G basis set was used, while the LANL2DZ basis set was applied for the palladium-containing MOF. Molecular structures were drawn and visualized using GaussView 5.0.8, and all quantum chemical calculations were carried out using Gaussian 09 Rev. A.02^68^. In addition, molecular docking studies were conducted using MOE 2008 (Molecular Operating Environment) software. The interaction between the MOF and selected proteins 3BE2, 7P16, 6ZX7, 5W63, 2W9Z, 1TUP and 4O2B was modeled using protein structures obtained from the Protein Data Bank (PDB). Molecular docking investigations were performed using Molecular Operating Environment (MOE) 2008 to evaluate the binding interactions of the monomeric and polymeric Pd-based pyrazine complexes with different biological targets. The crystal structures of VEGFR2 kinase, cleaved Caspase-3, Bcl-2, BAX, CDK4, p53, and the colchicine binding site of tubulin were retrieved from the Protein Data Bank using the PDB codes: 3BE2, 7P16, 6ZX7, 5W63, 2W9Z, 1TUP, and 4O2B, respectively. Prior to docking, all protein structures were prepared by removing water molecules, and non-essential ions, followed by the addition of hydrogen atoms and energy minimization using the MMFF94x force field. The active sites were defined based on the coordinates of the native co-crystallized ligands present in each protein structure. To validate the docking protocol, redocking of the native co-crystallized ligands into their corresponding active sites was performed. Prior to docking, the crystallographic structure of the synthesized 1D Pd-MOF was geometry optimized using DFT calculations, and the optimized repeating unit was extracted as the docking ligand. It should be noted that the molecular docking calculations were performed using the crystallographic repeating unit of the 1D Pd-MOF as a representative structural fragment. Because conventional docking algorithms are primarily developed for molecular ligands, the obtained docking results should be considered qualitative and exploratory, providing mechanistic support for the experimental biological findings rather than definitive evidence of the binding mode.

#### Table S2. Some of the optimized bond lengths, Å and bond angles, degrees, Pyz linker and its Pd(II) chain using B3LYP/6-311G and B3LYP/LANL2DZ respectively.

| Bond length (A^o^) | Pyz | Chain | Bond angles | Pyz | Chain |
| --- | --- | --- | --- | --- | --- |
| **R(Pd1-N5)** | --- | 2.09677 | **A(Cl14-Cl15-N5)** | 87.133 | 87.031 |
| **R(Pd1-Cl15)** | --- | 2.36265 | **A(N5-Pd1-N11)** | 93.601 | 93.425 |
| **R(C2-C3)** | 1.39483 | 1.40263 | **A(N4-Pd23-N27)** | --- | 179.737 |
| **R(C3-N5)** | 1.33484 | 1.36103 | **A(Cl32-Pd23-Cl33)** | --- | 179.064 |
| **R(N5-C6)** | 1.33490 | 1.36098 |  |  |  |
| **R(C2-N4)** | 1.33484 | 1.36005 |  |  |  |
| **R(N4-C7)** | 1.33490 | 1.36088 |  |  |  |

#### Table S3. Comparison of binding affinity of Pd-MOF against VEGFR2 kinase, cleaved Caspase 3, Bcl-2, BAX, CDK4, P53 and Colchicine Site of Tubulin using (Code: 3BE2, 7P16, 6ZX7, 5W63, 2W9Z, 1TUP and 4O2B, respectively) with Root Mean Square Deviation values (RMSD).

| **Docking results for monomer and polymer, 5-Fluro uracil, validated molecule, respectively** | | | |
| --- | --- | --- | --- |
| **Enzymes** | **Scoring Energy** **(kcal/mol) (RMSD)**  **Cis** | **Involved amino acids**  **(Atom bonding)** | **Type of interaction**  **(MOF atom bonding)** |
| **VEGFR2** | -4.79(2.08) | Glu-*885*  Asp-*1046* | Metal contact |
|  | -11.46(2.03) | Glu-*885*  Glu-*815* | Metal contact |
|  | -3.91(0.72) | Asp-1046, Phe-1047, Lys-868 | backbone acceptor and donor,  arene-H, sidechain donor |
| **N-{3-[3-(DIMETHYLAMINO)PROPYL]-5-(TRIFLUOROMETHYL)PHENYL}-4-METHYL-3-[(3-PYRIMIDIN-4-YLPYRIDIN-2-YL)AMINO]BENZAMIDE (**[**RAJ**](https://www.rcsb.org/ligand/RAJ)**)** | -5.05(2.62) | Gly-1048 | arene-H |
| **Capase-3** | -3.85(2.0) | Glu-156,Gln-37 | Metal Contact, Sidechain donor |
|  | -9.18(2.75) | Glu-156 | Metal Contact |
|  | -3.33(1.07) | Tyr-152 | Sidechain donor |
| **DIUNDECYL PHOSPHATIDYL CHOLINE (**[**PLC**](https://www.rcsb.org/ligand/PLC)**))** | -7.79(1.91) | (Phe-269, Trp-239), Cys-50 | arene-H, Sidechain acceptor |
| **Bcl-2** | -3.99(1.23) | DG-*10* | Metal contact |
|  | -10.42(2.57) | DG-*9*, DG-*10*, DA-*11* | Metal contact |
| **BAX** | -2.95(1.96) | Met-76 | Backbone acceptor |
|  | -5.81(2.70) | Ala-83 | arene-H |
|  | -2.91(1.58) | Arg-108, Asn-105 | Sidechain donor |
| **1,2-ETHANEDIOL** | -3.13(1.67) | Glu-44 | backbone acceptor |
| **CDK4** | -2.82 (2.56) | Glu-69,Glu-75 | Metal contact and Sidechain acceptor, arene-H |
|  | -8.92(2.9) | Glu-*69*, Glu-*75* | Metal contact, backbone donor |
| **P53** | -1.51(2.90) | Arg-*D248* | Sidechain donor |
|  | -3.19(1.5) | Glu--287 | Sidechain acceptor |
| **Colchicine Site of Tubulin** | -3.47(1.93) | Glu-183 | Metal Contact |
|  | -6.23(2.38) | Glu-183, Ser-178,Asn-206 | Sidechain acceptor, Backbone acceptor, arene-H |
|  | -3.65(1.10) | Val-177, Asn-206 | Backbone acceptor, Sidechain donor |
| **GUANOSINE-5'-TRIPHOSPHATE(**[**GTP**](https://www.rcsb.org/ligand/GTP)**)** | -7.03(2.27) | Gln-11, Asn-101, Ser-140, (Glu-71, Asp-98) | Backbone donor, Sidechain acceptor, Sidechain donor, Metal contact |
